# YOLOv5-POS: research on cabbage pose prediction method based on multi-task perception technology

**DOI:** 10.3389/fpls.2024.1455687

**Published:** 2024-09-23

**Authors:** Xiaojun Shen, Chaofan Shao, Danyi Cheng, Lili Yao, Cheng Zhou

**Affiliations:** ^1^ School of Information Engineering, Huzhou University, Huzhou, China; ^2^ Zhejiang Province Key Laboratory of Smart Management & Application of Modern Agricultural Resources, School of Information Engineering, Huzhou University, Huzhou, Zhejiang, China

**Keywords:** multi-task perception network, cabbage harvest, YOLOv5-POS, Bezier curve, posture recognition

## Abstract

**Introduction:**

Accurate and rapid identification of cabbage posture is crucial for minimizing damage to cabbage heads during mechanical harvesting. However, due to the structural complexity of cabbages, current methods encounter challenges in detecting and segmenting the heads and roots. Therefore, exploring efficient cabbage posture prediction methods is of great significance.

**Methods:**

This study introduces YOLOv5-POS, an innovative cabbage posture prediction approach. Building on the YOLOv5s backbone, this method enhances detection and segmentation capabilities for cabbage heads and roots by incorporating C-RepGFPN to replace the traditional Neck layer, optimizing feature extraction and upsampling strategies, and refining the C-Seg segmentation head. Additionally, a cabbage root growth prediction model based on Bézier curves is proposed, using the geometric moment method for key point identification and the anti-gravity stem-seeking principle to determine root-head junctions. It performs precision root growth curve fitting and prediction, effectively overcoming the challenge posed by the outer leaves completely enclosing the cabbage root stem.

**Results and discussion:**

YOLOv5-POS was tested on a multi-variety cabbage dataset, achieving an F1 score of 98.8% for head and root detection, with an instance segmentation accuracy of 93.5%. The posture recognition model demonstrated an average absolute error of 1.38° and an average relative error of 2.32%, while the root growth prediction model reached an accuracy of 98%. Cabbage posture recognition was completed within 28 milliseconds, enabling real-time harvesting. The enhanced model effectively addresses the challenges of cabbage segmentation and posture prediction, providing a highly accurate and efficient solution for automated harvesting, minimizing crop damage, and improving operational efficiency.

## 1 Introduction

The predominant method for cabbage harvesting today is mechanical harvesting, which offers advantages such as high efficiency and reduced labor costs. However, this method is also associated with a high incidence of mechanical damage (Ogedengbe et al., 2022). This damage primarily results from the diverse root shapes and complex postures of cabbages, further exacerbated by the high speed of mechanical operations. Consequently, it becomes challenging for the harvester to swiftly and accurately identify and assess the posture of the cabbage, and adjust the cutting position accordingly. Moreover, collisions and transmission vibrations among cabbage plants during the harvesting process can further disrupt the alignment of the cutting device (Tong et al., 2023). Therefore, achieving rapid and precision recognition of cabbage posture during harvesting is of significant research importance (Dongdong et al., 2015).

Detecting and localizing the cabbage root serves as a prerequisite for recognizing its posture. In previous research, the root recognition of conventional fruits and vegetables has typically relied on traditional image processing methods rooted in machine learning (Zhaoxin et al., 2022). For instance, Ying et al. (2000) proposed a straightforward algorithm based on Fourier descriptor technology to detect pear stems in traditional image processing. Luo et al. (2018) employed a segmentation algorithm based on k-means clustering and HSV color space to identify grape cluster pedicels and determine appropriate cutting points for each cluster. Xiong et al. (2018) utilized an improved fuzzy clustering method (FCM) and Otsu to segment images of lychee fruits and stems, achieving accurate calculation of the picking point for lychees during nighttime. Yamamoto et al. (2014) focused on tomato segmentation, distinguishing fruit, leaf, stem, and background based on pixel and blob information and concluded by employing X-means clustering to precisely detect individual intact tomato fruits on the plant. However, traditional image processing methods often face limitations in extracting high-dimensional information, making them susceptible to environmental illumination and object occlusion. Consequently, these factors contribute to reduced recognition accuracy when dealing with complex scenes involving fruit and vegetable roots (Hua et al., 2023).

Compared to traditional image processing methods, deep learning models possess enhanced capabilities in extracting high-dimensional features and end-to-end learning. This enables them to swiftly adapt to large-scale data, effectively addressing challenges posed by illumination variations, complex environments, and high-dimensional information extraction. As a result, deep learning models can significantly enhance the accuracy and stability of root recognition tasks (Sun et al., 2023; Roy and Bhaduri, 2022; Yu et al., 2019). For instance, Sa et al. (2016) utilized Faster R-CNN to integrate multi-modal (RGB and NIR) information, achieving precision separation of leaves and stems for various fruits (such as bell pepper, rock melon, strawberry, and apple) in complex environments. Wu et al. (2021) employed an improved YOLOv3 model optimized through clustering. This model demonstrated exceptional performance in rapidly and accurately identifying banana fruits, inflorescence axes, and flower buds, even under different lighting conditions. Zhu et al. (2023) proposed a method for detecting and locating tea buds based on YOLOv5s and 3D point clouds. They reconstructed YOLOv5s using the Efficient Channel Attention Network (ECANet) module and the Bidirectional Feature Pyramid Network (BiFPN), and combined it with the Density-Based Spatial Clustering of Applications with Noise (DBSCAN) clustering algorithm to achieve precision detection and localization of tea buds. Although the aforementioned method performs excellently in terms of detection accuracy, they were evaluated under unobstructed conditions, which limits their applicability in practical agricultural production. Therefore, the challenges of recognition caused by occlusion remain a key issue in current research.

According to existing reports, the combination of deep learning with image processing or machine learning methods has shown promising results in fruit recognition under partial occlusion. For example, Zhao et al. (2023) proposed a single-stage instance segmentation model that incorporates Deformable Convolutions (DCN) and the Convolutional Block Attention Module (CBAM) to classify peach images of nine different varieties and three maturity stages, even under complex conditions involving leaf occlusion or overlap. The model achieved a mean Average Precision (mAP) of 72.12%. Hussain et al. (2023) employed the Mask R-CNN algorithm to segment apple fruits and stems and used Principal Component Analysis (PCA) to estimate the orientations of the fruits and stems. This method successfully identified fruits and stems partially obscured by leaves, achieving average precision (AP) scores of 83.4% for fruits and 38.9% for stems. Sapkota et al. (2024) successfully implemented the YOLOv8 instance segmentation algorithm to achieve precision multi-class segmentation of trunks, branches, and unripe green fruits in the complex environment of an apple orchard, attaining a single-class instance segmentation mAP of 90.2% and a multi-class instance segmentation mAP of 74%. However, unlike the aforementioned studies, the connection point between the cabbage head and the root is completely obscured by outer leaves, making the root cutting position nearly impossible to observe. Additionally, the high computational demands and lower segmentation accuracy of existing research present difficulties in meeting the real-time requirements for mechanical harvesting.

In conclusion, given the diversity and morphological complexity of cabbage varieties, existing models face challenges in identifying the cut points of cabbage roots that are completely covered by outer leaves. Therefore, this study proposes a multi-task perception network based on the YOLOv5s architecture, named YOLOv5-POS, which optimizes the semantic segmentation head and neck layers to enhance model robustness. Additionally, a root growth prediction model based on Bézier curves was introduced to accurately predict the tilt angle of the cabbage root. The model achieves a mAP of 93.5% in instance segmentation, an average absolute error of 1.38° in posture prediction, an average relative error of 2.32°, and a detection time of 0.028 seconds. Compared with mainstream instance segmentation models such as YOLOv5s, YOLOv8n, and Mask-RCNN, as well as angle prediction methods like the minimum bounding rectangle and skeleton extraction, YOLOv5-POS demonstrates good performance.

The primary contributions of this study can be summarized as follows:

- Construction of a novel cabbage dataset: A comprehensive dataset comprising images of various cabbage varieties was meticulously curated. The root morphological structures of these varieties were classified into three main types: curved, short, and straight.
- Development of the YOLOv5-POS multi-task network: Based on the YOLOv5s framework, the YOLOv5-POS model was developed to simultaneously perform target detection and region segmentation of cabbage heads and roots. This was achieved by integrating the C-RepGFPN module within the Neck layer and optimizing the segmentation head. These enhancements enable effective refinement and fusion of high-level semantic features with low-level spatial features, thereby significantly enhancing detection accuracy.
- Establishment of a cabbage pose prediction model based on the Bezier curve: By combining the YOLOv5-POS multi-task network with Bezier curve fitting, this model enables precision determination of cabbage root poses and predicts their growth paths in complex occlusion scenarios. Additionally, the introduction of the KD-Tree algorithm accelerates the key point search process, significantly improving the detection speed of the model.

## 2 Materials and methods

### 2.1 Data sources

**Figure 1:**
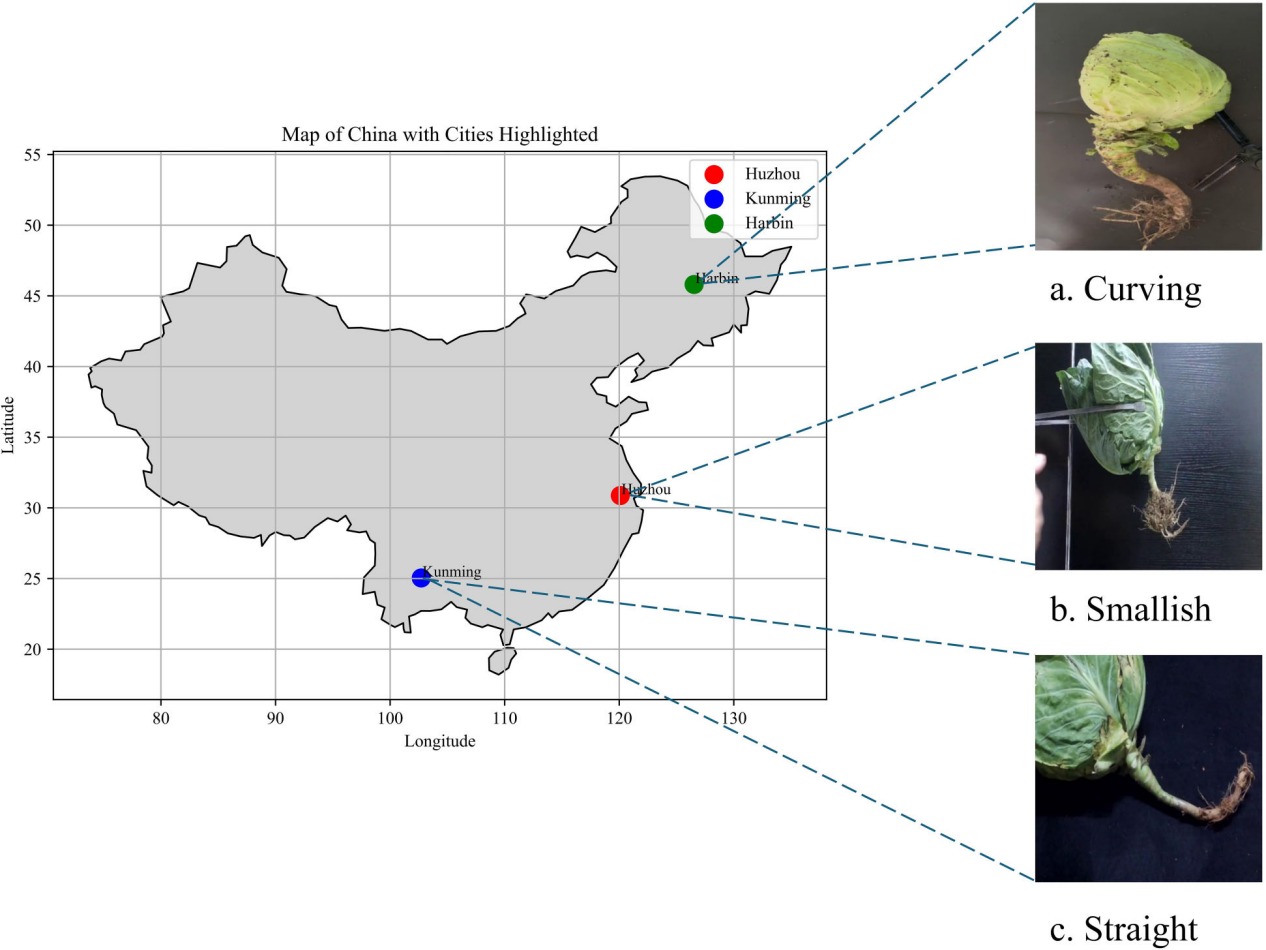
Collection locations and cabbage varieties from each region. **(A)** curving, **(B)** smallish, **(C)** Straight.

To capture the growth variations among cabbage varieties, plants from Kunming in Yunnan Province, Harbin in Heilongjiang Province, and Huzhou in Zhejiang Province were selected for this study. These plants showcased a spectrum of root shapes, encompassing curved, short, and straight varieties, as illustrated in **Figure 1**. Cabbages were secured with a clamping device, and images were captured using a CCD camera with a resolution of 1080×720 pixels positioned 30-40 cm from the cabbage. The images were collected from November to December 2022, resulting in a total of 988 images in different poses. Subsequently, the widely used annotation tool, Labelme, was employed to manually annotate cabbage heads and roots in each image. Target objects were delineated polygonally to exclude irrelevant pixels from the annotations. Of the entire dataset, 837 images were designated for training and validation, while 151 images were set aside for testing purposes.

### 2.2 Dataset preparation

**Figure 2:**
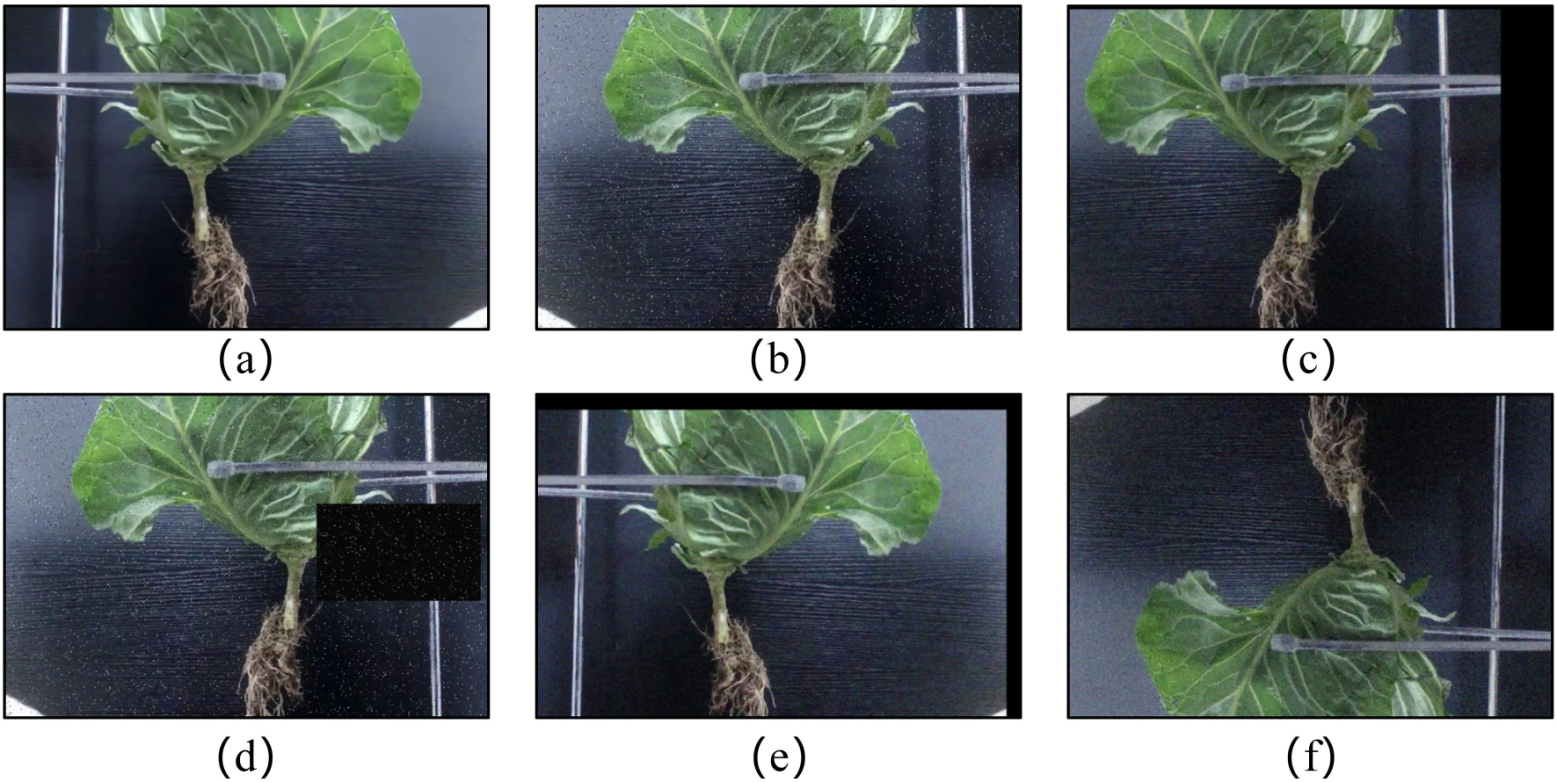
Image augmentation techniques applied. **(A)** Original image, **(B)** Mirror and noise, **(C)** Mirror and translate, **(D)** Flip with occlusion, **(E)** Translate and adjust brightness, **(F)** Mirror and flip.

To enhance the generalization capabilities of the model for real-world scenarios involving occlusion and low lighting conditions, image augmentation techniques were applied using the OpenCV library in Python. These techniques included random combinations of rotation, translation, brightness adjustments, noise addition, and increased occlusion, as depicted in **Figure 2**.

Each original image produced six new images, resulting in a total of 5022 images. However, it is important to note that data augmentation may impact the quality of some images. Therefore, a manual selection process was employed to retain an effective dataset, totaling 4912 images. Among these, 3930 images were used for training, while 982 images were allocated for validation.

### 2.3 Cabbage root posture and inclination recognition method

**Figure 3:**
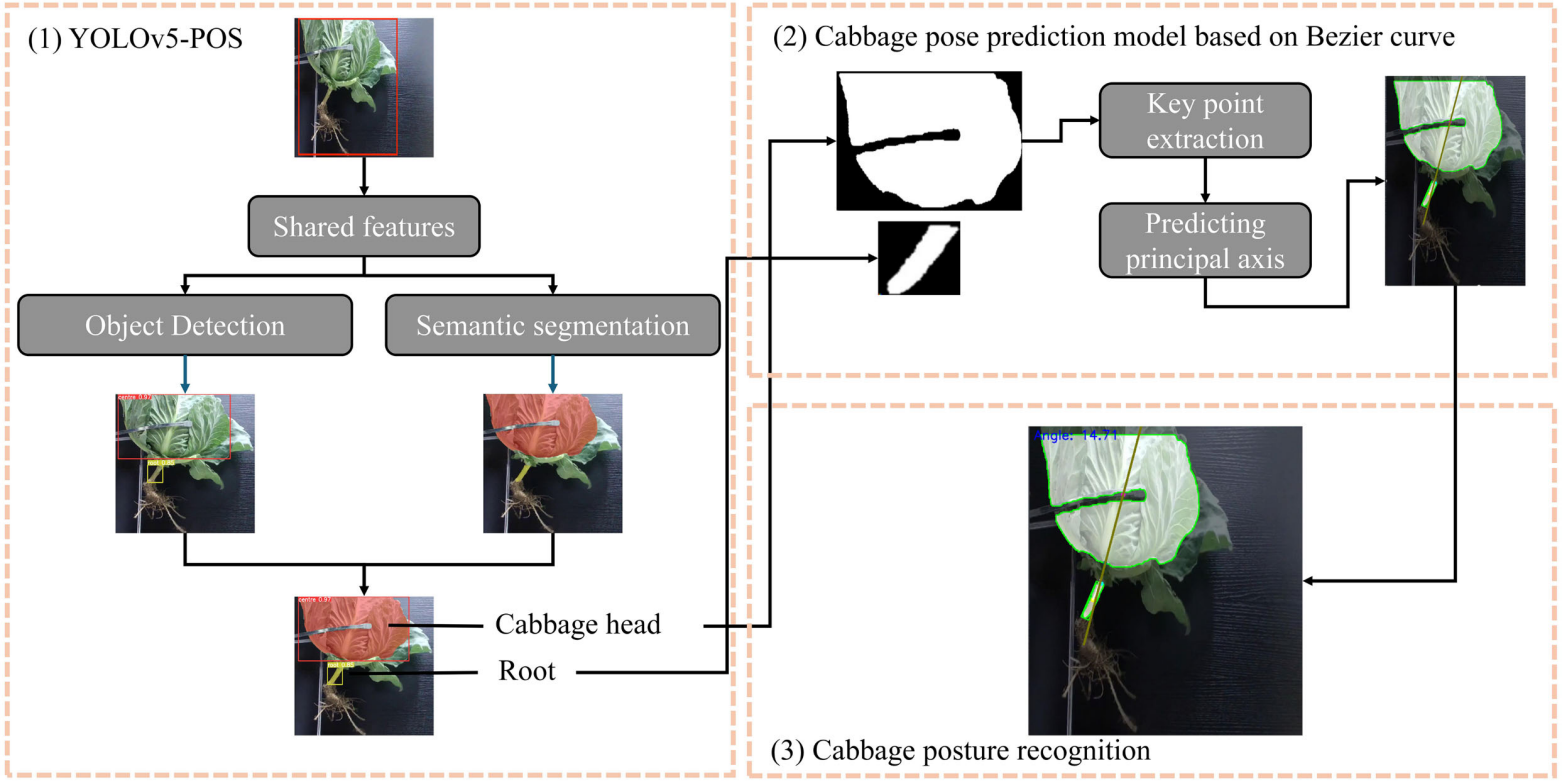
Cabbage posture prediction method based on YOLOv5-POS multi-task perception network.

As depicted in **Figure 3**, the proposed method for cabbage posture and inclination recognition, named YOLOv5-POS, consists of three main steps:

1. YOLOv5-POS Model Integration: Cabbage images are fed into a pre-trained YOLOv5-POS model to detect and segment cabbage heads and roots.
2. Bezier Curve and Key Point Extraction: Traditional image processing techniques are employed to extract key points, which are further identified using geometric moments and the minimum bounding rectangle method. Three key points are extracted: the centroid of the cabbage root, the nearest point at the root break, and the centroid of the cabbage head. These key points serve as control points for fitting the Bezier curve, which simulates the growth trajectory of the root. By connecting these key points on the cabbage, the main axis of the cabbage is determined. This method combines the precise description of geometric moments with the smooth fitting of Bezier curves, accurately depicting the shape and direction of the cabbage root.
3. Posture and Inclination Calculation: Using the predicted main axis of the cabbage, the angle between this axis and the ground is calculated to determine the cutting angle.

### 2.4 YOLOv5-POS multi-task network architecture

A multi-task learning network in deep learning refers to an advanced neural network architecture designed to simultaneously process and learn multiple interconnected tasks. This architecture typically consists of a shared encoder and several task-specific feature decoders (Lee and Seok, 2023). The shared layer, positioned at the lowest level of the network, extracts common features that are advantageous for all tasks. This capability enables the network to capture shared information across different tasks, thereby improving data utilization efficiency and the generalization capabilities of the model.

**Figure 4:**
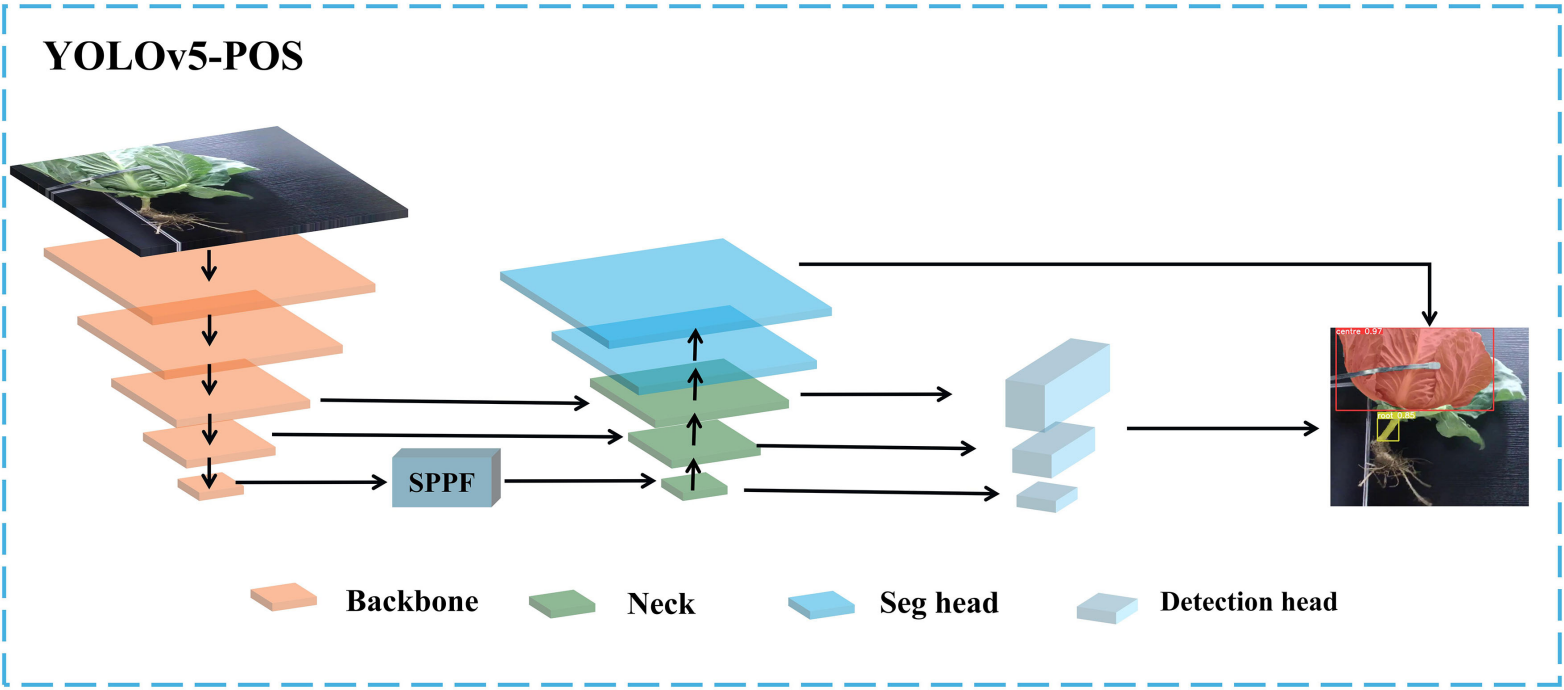
Multi-task architecture of YOLOv5-POS.

Taking advantage of the high accuracy and real-time performance of the YOLOv5s model, this study proposes a real-time multi-task convolutional neural network, named YOLOv5-POS, for the detection and segmentation of cabbage heads and roots. The network features a shared encoder and two independent decoders, as illustrated in **Figure 4**. The proposed multi-task network uses the existing Backbone and Neck layers as the shared encoder, while optimizing the Neck and Head layers. To minimize computational overhead and maintain the one-stage detector structure, redundant shared blocks between different decoders are omitted.

#### 2.4.1 The overall structure of YOLOv5s

The YOLO series algorithms have gained significant popularity in the field of computer vision object detection due to their remarkable accuracy and real-time performance. Through continuous optimization and iteration, these algorithms have evolved to the YOLOv10 version. However, despite the improved performance metrics in the latest iteration, it often requires substantial computing resources and memory, which can hinder detection speed and model deployment.

To meet the requirements of the cabbage posture recognition, focusing on detection speed and lightweight model deployment, this study utilizes the YOLOv5s model as the foundation for improvement. The model size of YOLOv5s is a mere 14.1 MB (Liu et al., 2023; Tian et al., 2023). The YOLOv5s network structure can be divided into three parts: backbone, neck, and head. The backbone primarily extracts basic features from the image and typically comprises Conv (Convolutions), C3 (Cross Stage Partial Networks Bottleneck with 3 convolutions), and SPPF (Spatial Pyramid Pooling – Fast). This network captures rich information from the input image. The neck layer refines and fuses features extracted from the backbone using a Feature Pyramid Network (FPN) and a Path Aggregation Network (PAN), enhancing the ability of the model to recognize objects at different scales. The head is responsible for predicting and decoding the output from the neck to generate the categories and location information of the targets.

**Figure 5:**
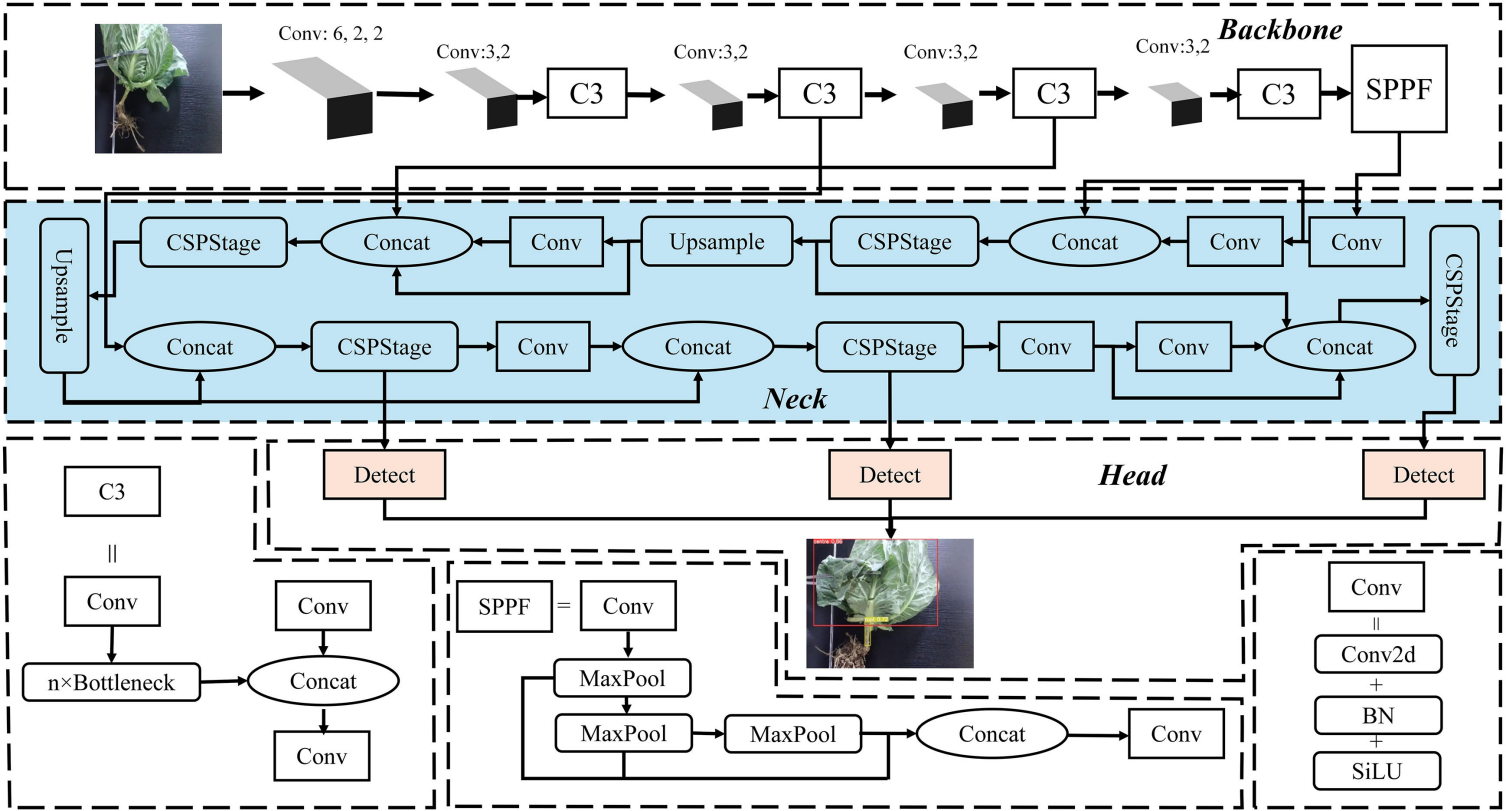
The structure of YOLOv5-POS model.

Building upon the fundamental structure of YOLOv5s, this study introduces an efficient multi-scale feature fusion module called C-RepGFPN, which leverages multi-layer aggregation and reparameterization techniques. This module enhances the interaction among features of different scales to accommodate changes in object proportions across different scenes. Moreover, the segmentation Head (C-seg) is improved based on the original Head layer, capitalizing on its ability to extract and fuse multi-scale features. This enhancement improves adaptability to changes in target scale and enhances the ability of the model to capture intricate details. The structure of the improved model is illustrated in **Figure 5**.

#### 2.4.2 C-RepGFPN neck

**Figure 6:**
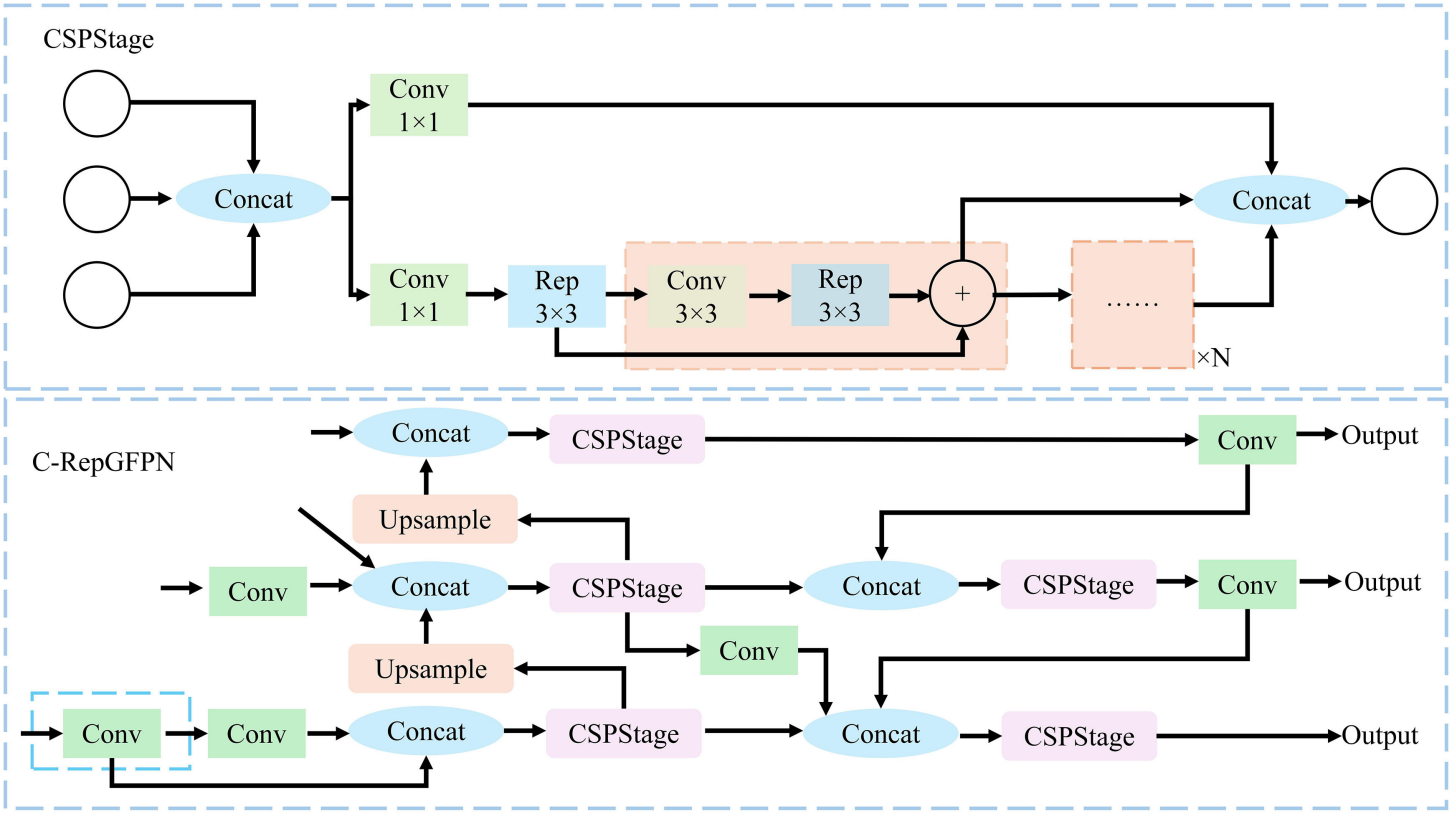
Diagram of the C-RepGFPN Neck network structure.

To enhance the performance of the YOLOv5s model in the cabbage segmentation task, particularly its ability to varying cabbage sizes in different scenarios, this study introduces the C-RepGFPN Neck. This incorporates the Efficiency-RepgFPN architecture (Wang et al., 2023), optimizing the structure and fusion method of the Feature Pyramid Network (FPN). This enhancement effectively reduces redundant computing and memory consumption. Additionally, the introduction of the re-parameterization mechanism and ELAN into the CSPNet branch enhances feature interaction across different scales and improves the quality of feature fusion. Building on these optimizations, efficiency in feature extraction is further enhanced by integrating a 1x1 convolution kernel into the Efficient-RepGFPN architecture. This reduces the dimensionality of feature maps extracted by the Backbone, facilitating streamlined CSPStage processing. These enhancements in the C-RepGFPN Neck not only improve feature extraction efficiency but also significantly enhance model accuracy in segmenting cabbages of various sizes and shapes. The specific structure and operational principles of the C-RepGFPN Neck are illustrated in **Figure 6**.

#### 2.4.3 C-Seg segmentation head

To tackle the challenges posed by diverse backgrounds, varying degrees of occlusion, and changes in illumination on cabbage recognition, this study proposes the C-Seg segmentation head model. This model combines the efficient detection capabilities of the YOLOv5s network with the exceptional performance of the U-Net model in image segmentation (Ronneberger et al., 2015). The objective is to enhance the ability of the model to capture edge and texture information of cabbage, thereby achieving more accurate recognition and segmentation while maintaining real-time performance.

**Figure 7:**
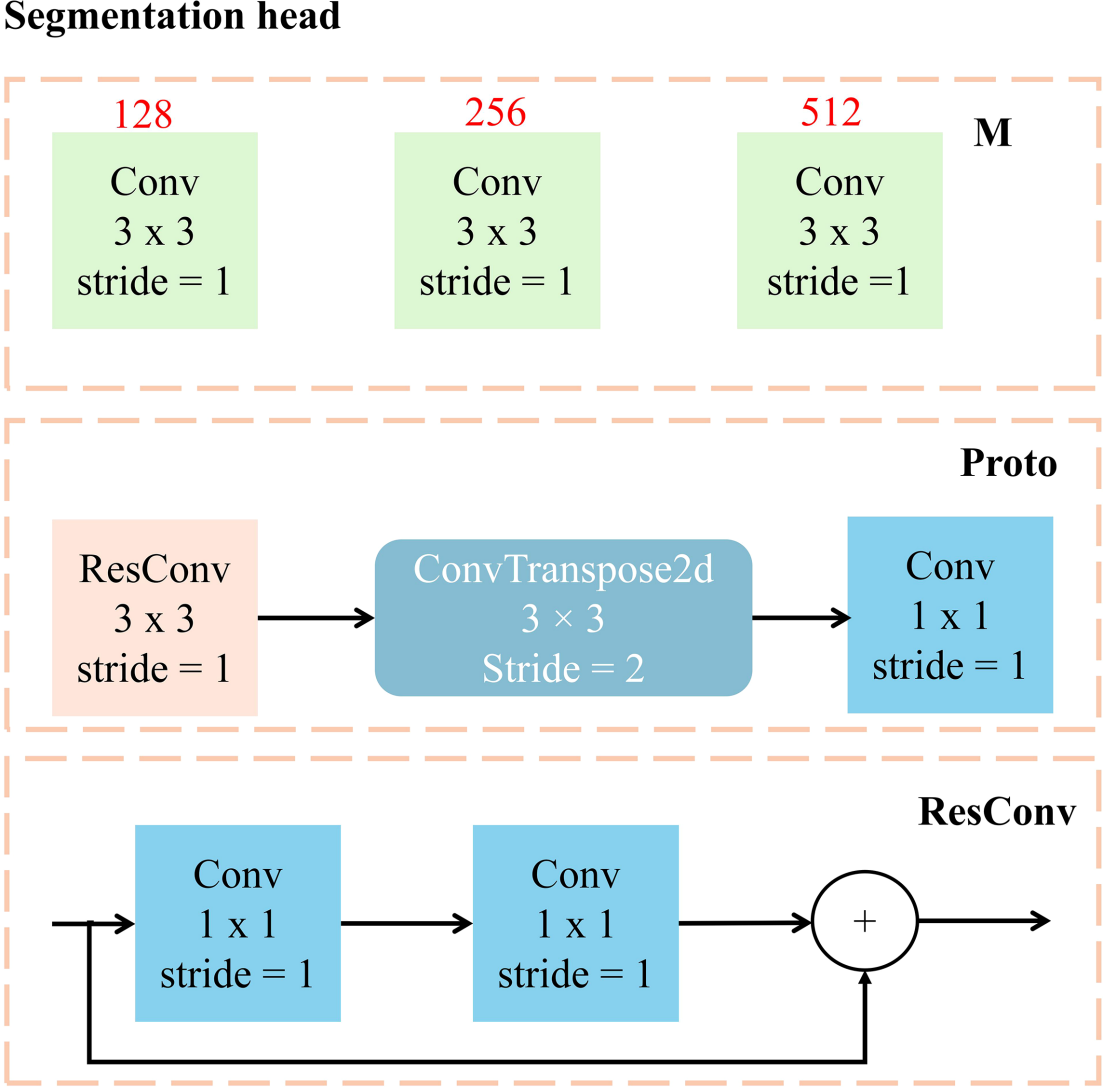
Workflow diagram of the detection head.

The C-Seg segmentation head consists of two main components: ModuleList and Proto, as depicted in **Figure 7**. The ModuleList performs convolution operations on feature maps of different scales using three convolution layers, extracting and enhancing scale-specific feature information. Conversely, the Proto component includes a residual convolutional layer, a convolutional layer, and a deconvolution layer. Together, these layers facilitate feature extraction, upsampling, and the generation of segmentation results. Notably, the residual convolutional layer improves feature transfer through residual connections, enhancing training stability and enabling the model to capture deeper features.

To further optimize the performance of the model, a ResConv convolutional layer incorporating residual connections replaces the standard convolutional layer in the original YOLOv5s segmentation head. This substitution effectively enhances the depth and feature expression capabilities of the network. Additionally, deconvolution operations are employed to achieve feature map upsampling and adjust the number of channels in the model. This strategy substantially reduces the number of model parameters and the consumption of computing resources, resulting in a more lightweight network structure.

### 2.5 Cabbage posture prediction model based on Bezier curve

**Figure 8:**
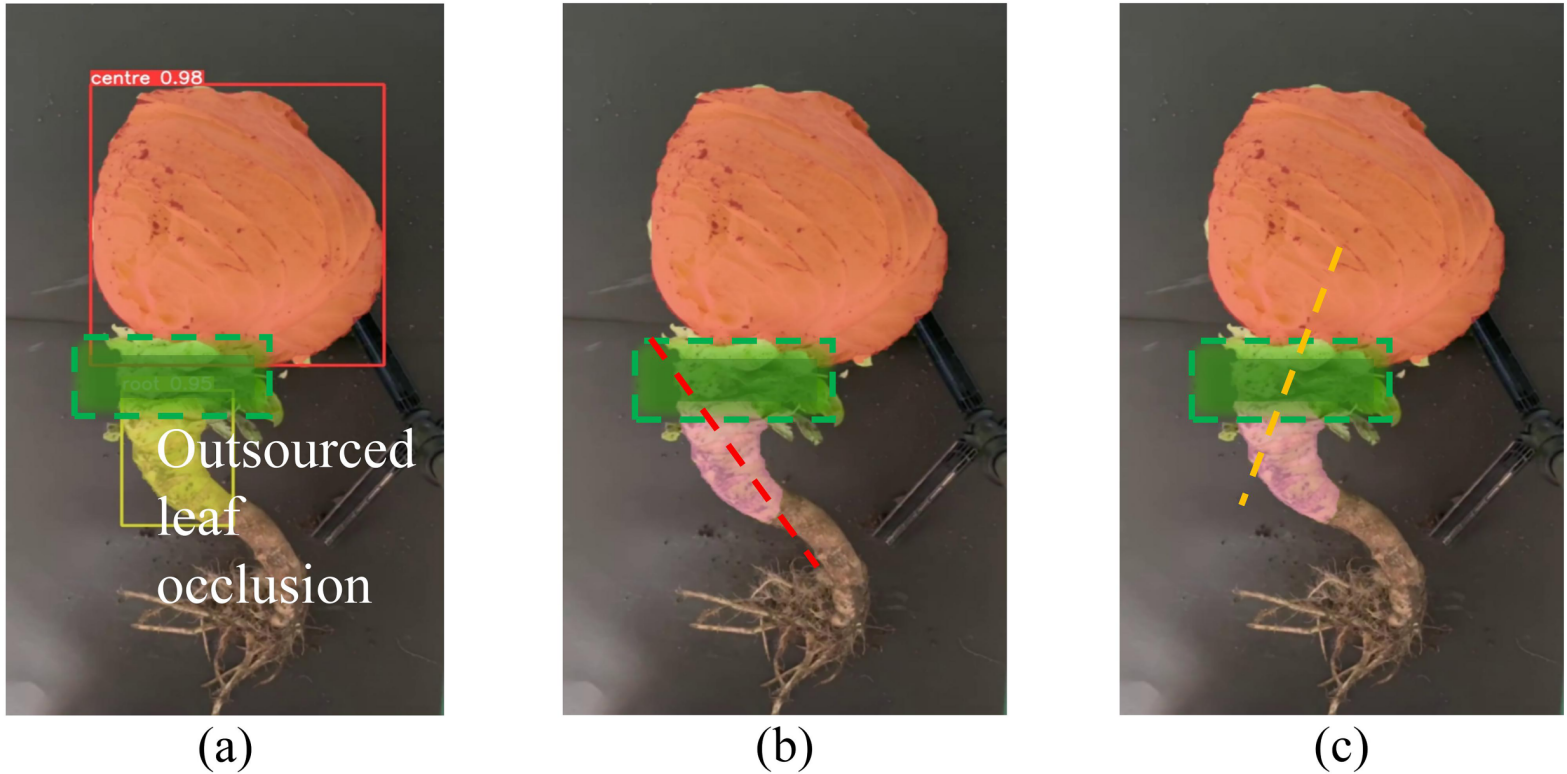
Schematic diagram of cabbage root inclination recognition error. **(A)** YOLOv5-POS recognition and segmentation results, **(B)** Mismeasurement results due to occlusion of outsourced leaves, **(C)** Actual measurement results.

In practical cultivation scenarios, cabbage roots often bend and are wrapped by outer leaves (Dai et al., 2016). **Figure 8A** illustrates a cabbage plant with its outer leaves removed, where the YOLOv5-POS model effectively identifies and segments the cabbage head and root. However, when a portion of the root is concealed by outer leaves, the visible growth area of the lower root is frequently misclassified as the actual growth direction. This results in erroneous detection, as indicated by the red dotted line in **Figure 8B**, whereas the true main axis of the cabbage root is depicted in in **Figure 8C**. Thus, relying solely on YOLOv5-POS for image recognition and segmentation does not adequately address the challenge of cabbage pose recognition.

Furthermore, the occlusion caused by the outer leaves obscures the connection point between the cabbage head and root after instance segmentation, complicating the ability of harvester to determine the precision cutting position. To tackle this issue, this study draws inspiration from anti-gravity stem tracking root image inpainting algorithm proposed by Mingxuan et al. (2022), originally developed for occluded maize roots. A Bessel curve fitting method is employed to create a prediction model that restores the integrity of the occluded area in the root.

**Figure 9:**
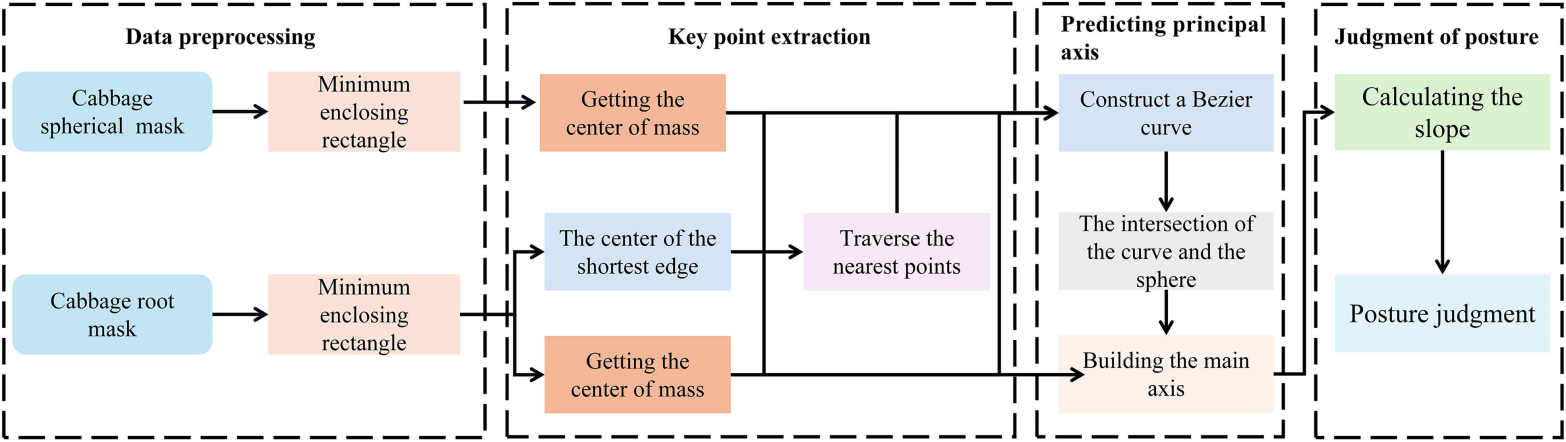
Flowchart of cabbage pose prediction model based on Bezier curve.

By combining this model with the YOLOv5-POS multi-task network, accurate posture judgment of the cabbage root is achieved. The specific judgment process, as depicted in **Figure 9**, involves four main steps: preprocessing of multi-task network outputs based on YOLOv5-POS, extraction of key points using the Bessel curve, development of a principal axis prediction model, and final posture assessment.

#### 2.5.1 Determination of the main axis of cabbage

**Figure 10:**
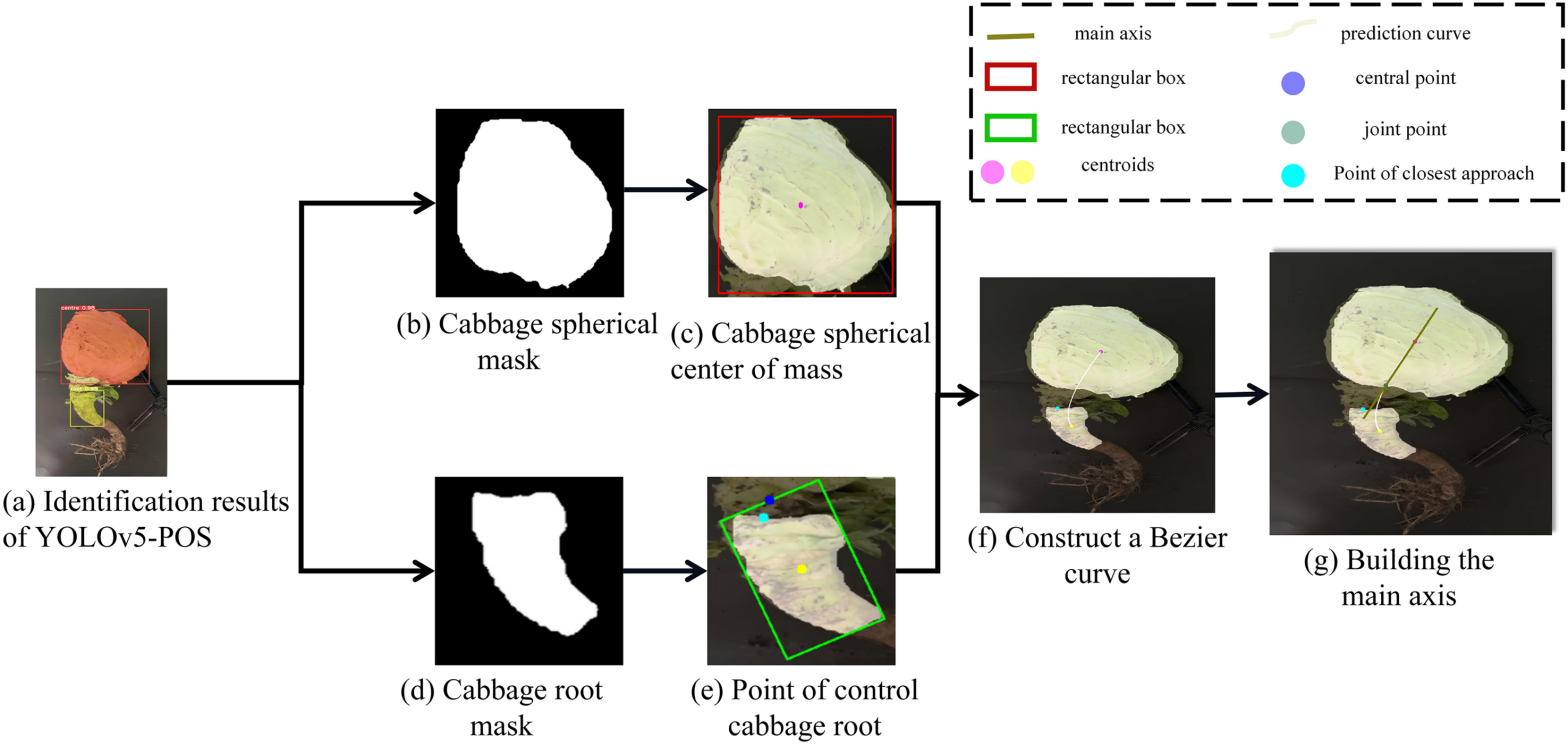
Flowchart of cabbage pose prediction model based on Bezier curve.

As illustrated in **Figure 10**, the YOLOv5-POS multi-task network model determines the positions of the cabbage root and head in the image. This method employs a geometric model to streamline the segmentation of the cabbage head and root target regions. Utilizing the geometric moment method, the model calculates the minimum bounding rectangle and centroid point of the cabbage head and root, facilitating the determination of their position and mass distribution. Subsequently, the closest breakpoint is identified by traversing the midpoint of the root mask from the uppermost edge of the minimum enclosing rectangle. Key points for the Bezier curve are determined based on the centroid point of the cabbage head, the centroid point of the cabbage root, and the closest point. The Bezier curve is then constructed to predict the growth path of the root. Finally, the principal axis of the cabbage is obtained by calculating the intersection point between the curve and the cabbage head mask, and connecting it with the centroid of the cabbage head.

#### 2.5.2 Determination of the Bessel curve control point

In this study, the Bezier curve representing the posture of the cabbage root is constructed using three key control points: the centroid point P_0_ of the cabbage root, the nearest point P_1_ to the breakpoint of the cabbage root, and the centroid point P_2_. The connection between the breakpoint and the cabbage head is determined based on the anti-gravity stem tracking principle of the plant, making P_1_ an essential vertex of the Bezier curve.

**Figure 11:**
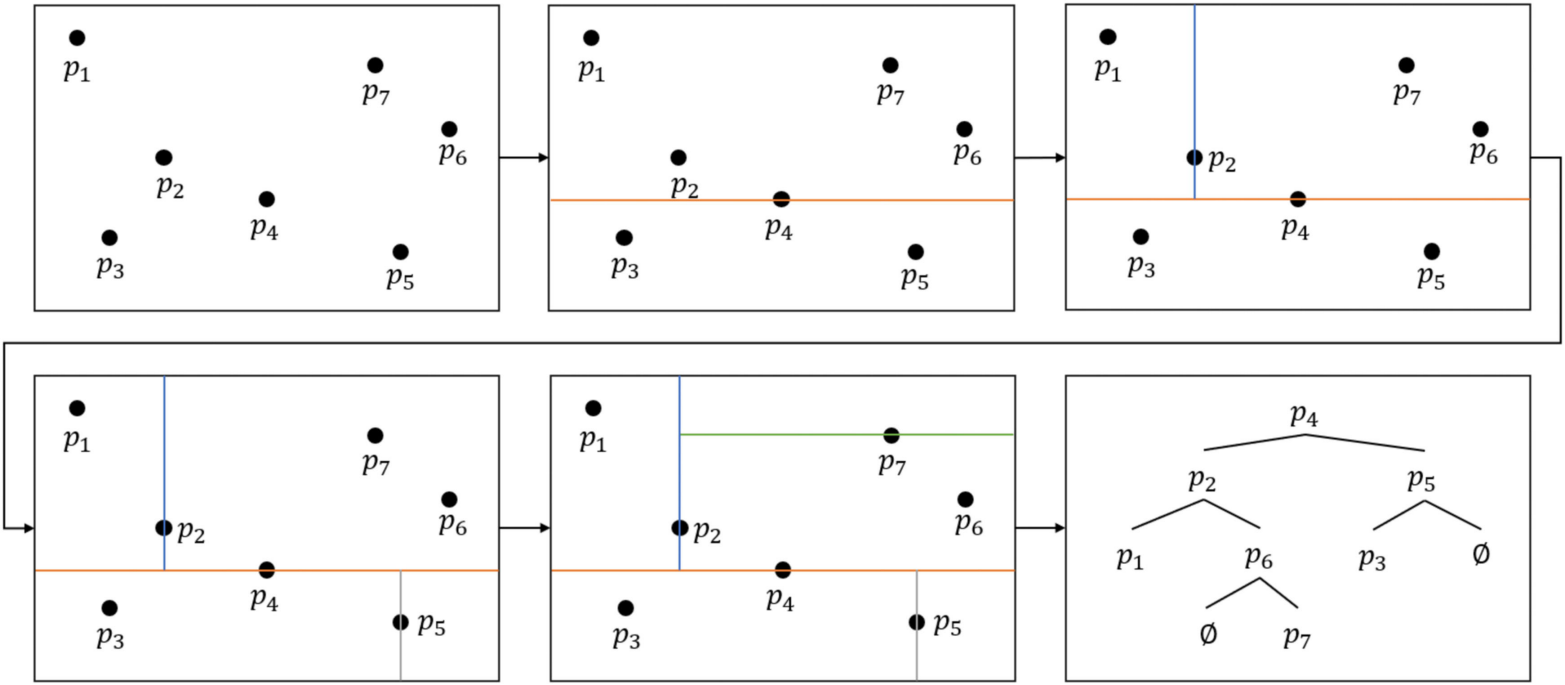
KD-Tree building process.


The search for the point P_1_ significantly influences the calculation speed of the prediction model. To expedite this search process, a KD-Tree method is employed to establish the topological relationship between the spline curve points and the root contour points. A KD-Tree is a specialized data structure that organizes k-dimensional data for rapid data retrieval. By recursively dividing the k-dimensional space, the KD-Tree efficiently organizes and retrieves data, excelling in tasks such as nearest neighbor search and range search for large-scale multi-dimensional data, ensuring efficient performance (Jin et al., 2023). **Figure 11** illustrates the construction process of the KD-Tree. Initially, a partition dimension and partition value are selected, typically choosing the median of all data points within the current dimension as the partition value. Subsequently, the data points are divided into two subsets based on this split value. This process is repeated on each subset until all data points are contained in the leaf nodes of the tree. Consequently, each node of the binary tree corresponds to a k-dimensional hyperrectangular region, effectively capturing the distribution and relationship of the data points in the k-dimensional space. The time complexity of the KD-Tree is as follows:

(2.1) $$\begin{array}{c} \text{T}\left(\text{n}\right)\{\begin{array}{c} \text{O}\left(1\right), n=1\\ \text{O}\left(\text{n}\right)+2\cdot \text{T}\left(\left[\frac{2}{\text{n}}\right]\right),n>1 \end{array} \end{array}$$

Bezier curves can be recursively generated and are defined by n+1 control points and n interpolation points for a Bezier curve of degree n. The first and last control points represent the starting and ending points of the curve, while the remaining control points are responsible for shaping the curve. The interpolation points serve to connect adjacent control points, creating a smooth and continuous curve. To calculate a point P(t) on a Bezier curve of degree n, the following formula is utilized:


(2.2) $$\text{P}\left(\text{t}\right)={\left(1-t\right)}^{\text{n}}{\times \text{P}}_{0}\text{+C}\left(n,1\right)\times \text{t}\times {\left(1-t\right)}^{\left(n-1\right)}{\times \text{P}}_{1}+\cdots \text{+C}\left(n,n-1\right){\times \text{t}}^{\left(n-1\right)}\times \left(1-t\right){\times \text{P}}_{\left(n-1\right)}{\text{+t}}^{\text{n}}{\times \text{P}}_{\text{n}}$$

Where t is a parameter ranging from 0 to 1, denoting the position along the curve. ${\text{P}}_{0}$ , ${\text{P}}_{1}$ , ⋯ , ${\text{P}}_{n}$ represent the n+1 control points, and C(n,i) denotes the binomial coefficient, given by $\frac{\text{n!}}{\left(\text{i!}\times \;\left(\text{n-i}\right)\right)}$ . Although this equation can be employed to compute points on the curve, the computational complexity is substantial, particularly for higher-order curves. Hence, the De Casteljau algorithm (Sanchez-Reyes, 2020) is utilized to calculate each linear combination through multiple recursive steps, ultimately yielding the desired points on the curve.

**Figure 12:**
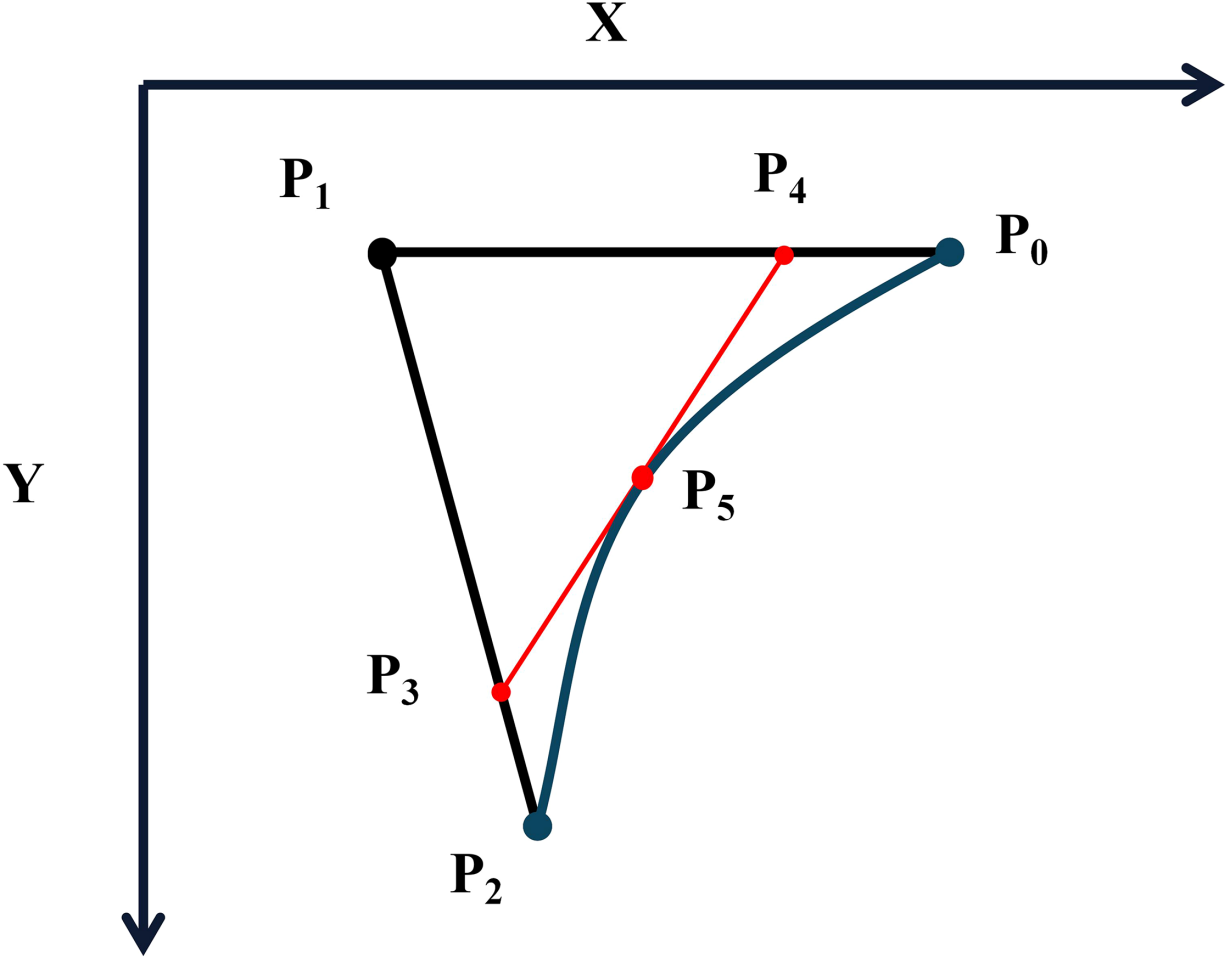
Bezier key point determination curve.


This relationship is depicted in **Figure 12**, where the line ${\text{P}}_{4}{\text{P}}_{5}{\text{P}}_{3}$ represents the tangent to the curve ${\text{P}}_{0}{\text{P}}_{5}{\text{P}}_{2}$ , with ${\text{P}}_{5}$ serving as the tangent point. To compute the points on the second-order Bezier curve using the De Casteljau algorithm, a recursive approach is employed, incorporating the parameter k. The algorithm is outlined as follows:

(2.3) $$\begin{array}{c} {\text{Q}}_{0}\text{ = }\left(1 - t\right){\times \text{P}}_{0}{\text{ + t}\times \text{P}}_{1} \end{array}$$

(2.4) $$\begin{array}{c} {\text{Q}}_{1}\text{ = }\left(1 - t\right){\times \text{P}}_{1}{\text{ + t}\times \text{P}}_{2} \end{array}$$

${\text{Q}}_{0}$ and ${\text{Q}}_{1}$ are linear interpolation points,


(2.5) $$\begin{array}{c} \text{B}\left(\text{t}\right)\text{= }\left(1 - t\right){\times \text{Q}}_{0}{\text{ + t}\times \text{Q}}_{1} \end{array}$$

When t ∈ [0,1], it represents a quadratic Bezier curve ${\text{P}}_{0}$ , ${\text{P}}_{1}$ , ${\text{P}}_{2}$ defined by the 3−vertices ${\text{P}}_{0}{\text{P}}_{2}$ .

#### 2.5.3 Cabbage posture judgment

In accordance with the conveying structure of the clamping conveying device on the cabbage harvester, its primary function is to facilitate the seamless transportation of cabbage to the cutting device. Throughout the conveying process, it is imperative to maintain the cabbage in a relatively immobile state, in perfect alignment with the clamping conveyor belt, thereby enabling the root cutting device to effectively sever the cabbage root. In order to ensure the precision of the root cutting process, it is vital to keep the cabbage root at an optimal angle within the designated cutting range. Any deviation beyond this range is deemed an oblique state. Consequently, the point of intersection between the projected root growth path, as determined by the Bezier curve, and the cabbage head mask, is denoted as ${\text{P}}_{3}\left(x_{3}\text{, }y_{3}\right)$ . The midpoint directly above it is denoted as ${\text{P}}_{2}\left(x_{2},\;y_{2}\right)$ , as visually depicted in **Figure 10G**. The slope formula is expressed as follows:


(2.6) $$\begin{array}{c} \theta =\left|tan^{-1}\left(\frac{y_{2}-y_{3}}{x_{2}-x_{3}}\right)\right| \end{array}$$

Compare the obtained θ with the cutting threshold Angle β to determine the cabbage pose:


(2.7) $$\begin{array}{c} \text{cabbage posture}\{\begin{array}{c} \text{Stay vertical, θ}\leq \text{β}\\ \text{Tilt of attitude, θ>β} \end{array} \end{array}$$

### 2.6 Model performance evaluation and experimental environment

#### 2.6.1 Model performance evaluation

This study conducts ablation experiments on multi-task networks and compares them with various commonly used multi-task networks and instance segmentation models to assess the performance of the YOLOv5-POS model. The primary evaluation metrics for the detection model include Recall, Precision, and mean Average Precision (mAP). Precision denotes the ratio of correctly identified targets to the total detected targets, while recall represents the proportion of true targets that are successfully detected. The F1 score, a harmonic mean of precision and recall, serves as an indicator of the overall stability and robustness of the model. A higher F1 score typically implies greater reliability. The F1 score is calculated using the following formula:


(2.8) $$\begin{array}{c} \text{Recall=}\frac{\text{TP}}{\text{TP+FN}} \end{array}$$

(2.9) $$\begin{array}{c} \text{Precision=}\frac{\text{TP}}{\text{TP+FP}} \end{array}$$

(2.10) $$\begin{array}{c} F1=\frac{2\times \text{Recall}\times \text{Precision}}{\text{Recall + Precision }} \end{array}$$

The calculation of the F1 score involves the utilization of TP (true positives), FP (false positives), and FN (false negatives). Specifically, TP represents the count of correctly identified examples, FP denotes the count of incorrectly identified positive examples, and FN signifies the count of missed positive examples.

In the context of evaluating instance segmentation models, the primary metric employed is the mAP. This metric is computed as follows:


(2.11) $$\begin{array}{c} AP=\int_{0}^{1}P\left(R\right)dR \end{array}$$

(2.12) $$\begin{array}{c} mAP=\frac{1}{n}\sum_{n}AP \end{array}$$

In the formula for mAP, P represents Precision and R represents Recall. Intersection over Union (IoU) is a measure of how well the predicted bounding box aligns with the ground truth bounding box, where larger values indicate more accurate predictions.

mAP provides a comprehensive assessment of the detection performance across various IoU thresholds. mAP@0.5 specifically emphasizes the approximate accuracy, while mAP@0.5:0.95 requires the model to perform well across multiple IoU thresholds. Additionally, the Frame rate (FPS) reflects the detection speed of the model.

#### 2.6.2 Test results evaluation metrics

In this study, three evaluation metrics were employed to compare the computational measurements of angles: Mean Absolute Error (MAE), Mean Squared Error (MSE), and Root Mean Squared Error (RMSE). Both MSE and MAE quantify the discrepancy between predicted and true values. MSE is the average of the squared differences between predictions and true values, whereas MAE is the average of the absolute differences. RMSE, derived as the square root of MSE, normalizes the squared errors to the same scale as the original values, offering a more interpretable measure of the prediction error. Its formula is as follows:


(2.13) $$\begin{array}{c} \text{MAE=}\frac{1}{\text{n}}\sum_{i=1}^{\text{n}}\left|{\text{y}}_{\text{r}}{\text{-y}}_{\text{p}}\right| \end{array}$$

(2.14) $$\begin{array}{c} \text{MSE=}\frac{1}{\text{n}}\sum_{i=1}^{\text{n}}{\left({\text{y}}_{\text{r}}{\text{-y}}_{\text{p}}\right)}^{2} \end{array}$$

(2.15) $$\begin{array}{c} \text{RMSE=}\sqrt{\frac{1}{\text{n}}\sum_{i=1}^{\text{n}}{\left({\text{y}}_{\text{r}}{\text{-y}}_{\text{p}}\right)}^{2}} \end{array}$$

Where, $y_{r}$ represents the true value, y_p_ represents the predicted value, and n represents the number of samples.

#### 2.6.3 Experimental environment and configuration

The Autodl server serves as the foundational platform for establishing the training environment, operating primarily on Ubuntu 20.04. PyTorch serves as the deep learning framework of choice for conducting a series of experiments. Hardware experiments were performed using an NVIDIA GeForce RTX 2080Ti, complemented by a cloud server with 11GB of memory.

For the initial training, YOLOv5-POS utilizes the model parameters outlined in **Table 1**. To expedite the training process, a pre-trained model from the YOLOv5 project is employed for transfer learning. Throughout training iterations, the network continually saves parameters associated with the highest accuracy achieved.

**Table 1:** The parameter setting of the YOLOv5-POS.

Parameters	Value
Learning rate	0.01
Momentum	0.937
Weight decay	0.0005
Epoch	100
Batch-size	8
Optimizer	98.4%

Furthermore, the MMDetection open-source object detection toolbox is utilized to compare the implementation of various object detection model algorithms, such as Mask R-CNN, Cascade R-CNN, SOLOv2, HTC, among others. All networks adhere to the same training environment settings and datasets, employing their default initialization parameters and pre-trained weights to ensure optimal network performance.

## 3 Analysis and results

### 3.1 Model training

**Figure 13:**
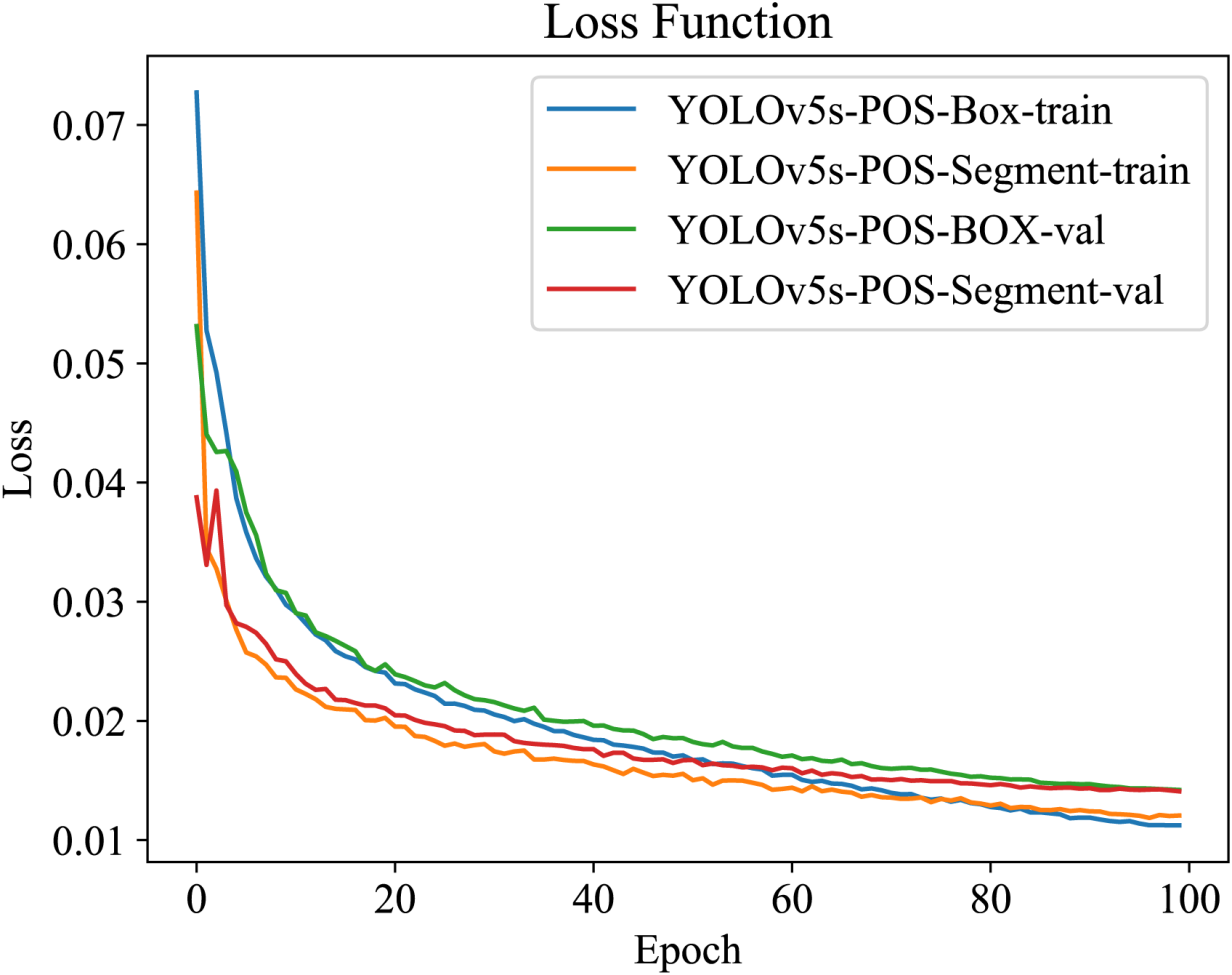
Model loss results.

For a comprehensive comparison, all enhanced models underwent training and validation using the same datasets. As depicted in **Figure 13**, the training loss steadily decreases with increasing iterations. Initially, training commenced with the provided weight file YOLOv5s.pt. Notably, the training loss curve exhibits rapid convergence within the first 20 iterations. Subsequently, the rate of loss reduction gradually diminishes, reaching a plateau around 60 iterations. Beyond approximately 100 iterations, both loss values and accuracy stabilize, displaying minimal fluctuations.

### 3.2 Ablation experiments

The efficacy of the model improvement was evaluated through ablation experiments to determine the impact of the enhanced components on the performance of the model. Building upon the original YOLOv5s algorithm, an improved method was incorporated at each stage, resulting in four sets of experimental comparisons. The outcomes are presented in **Table 2**.

**Table 2:** The results of the YOLOv5-POS on the Ablation studies.

Model	Box				Mask				Parameters	GFLOPs
	Precision	Recall	mAP@0.5	F1	Precision	Recall	mAP@0.5	mAP@0.5:0.95		
YOLOv5s	98.2%	89.7%	93.0%	98.4%	98.4%	89.4%	92.7%	79.9%	7.06	25.7
+C-RepGFPN	98.8%	89.8%	93.5%	98.8%	98.8%	89.7%	93.0%	80.1%	9.18	29.5
+C-Seg	97.9%	90.1%	92.7%	98.1%	98.1%	90.3%	92.7%	78.5%	6.98	21.5
YOLOv5-POS	97.5%	90.1%	93.5%	98.8%	98.8%	90.7%	93.3%	80.1%	9.10	25.1

The results presented in **Table 2** show that YOLOv5s-C-FGPN enhances accuracy, recall rate, and mAP@0.5 indicators for object detection and instance segmentation, with F1 showing a 0.4% improvement. The Mask segment shows a 0.2% increase in mAP@0.5:0.95, enabling superior image feature extraction and enhancing model accuracy and recall. However, the increased accuracy comes with a rise in model parameters, with GFPLOPs increasing from 25.7 to 29.5.

In contrast, the Box target detection and Mask image segmentation components of YOLOv5-C-Seg exhibit slight decreases in accuracy and mAP@0.5, with F1 and mAP@0.5:0.95 declining by 0.3% and 1.4% respectively. However, model recall improves by 0.4% and 0.9%, indicating better detection of true targets. Notably, YOLOv5-Seg reduces the number of parameters, with GFLOPs decreasing from 25.7 to 21.5, thus improving computational efficiency.

Regarding the YOLOv5-POS model, it exhibits an increase of 0.3 percentage points in mAP for object detection and 0.8 percentage points in image segmentation, signifying enhanced accuracy. Despite the increase in model parameters, the proposed model effectively reduces GFLOPs while optimizing calculations, showcasing improved computational optimization capabilities.

**Figure 14:**
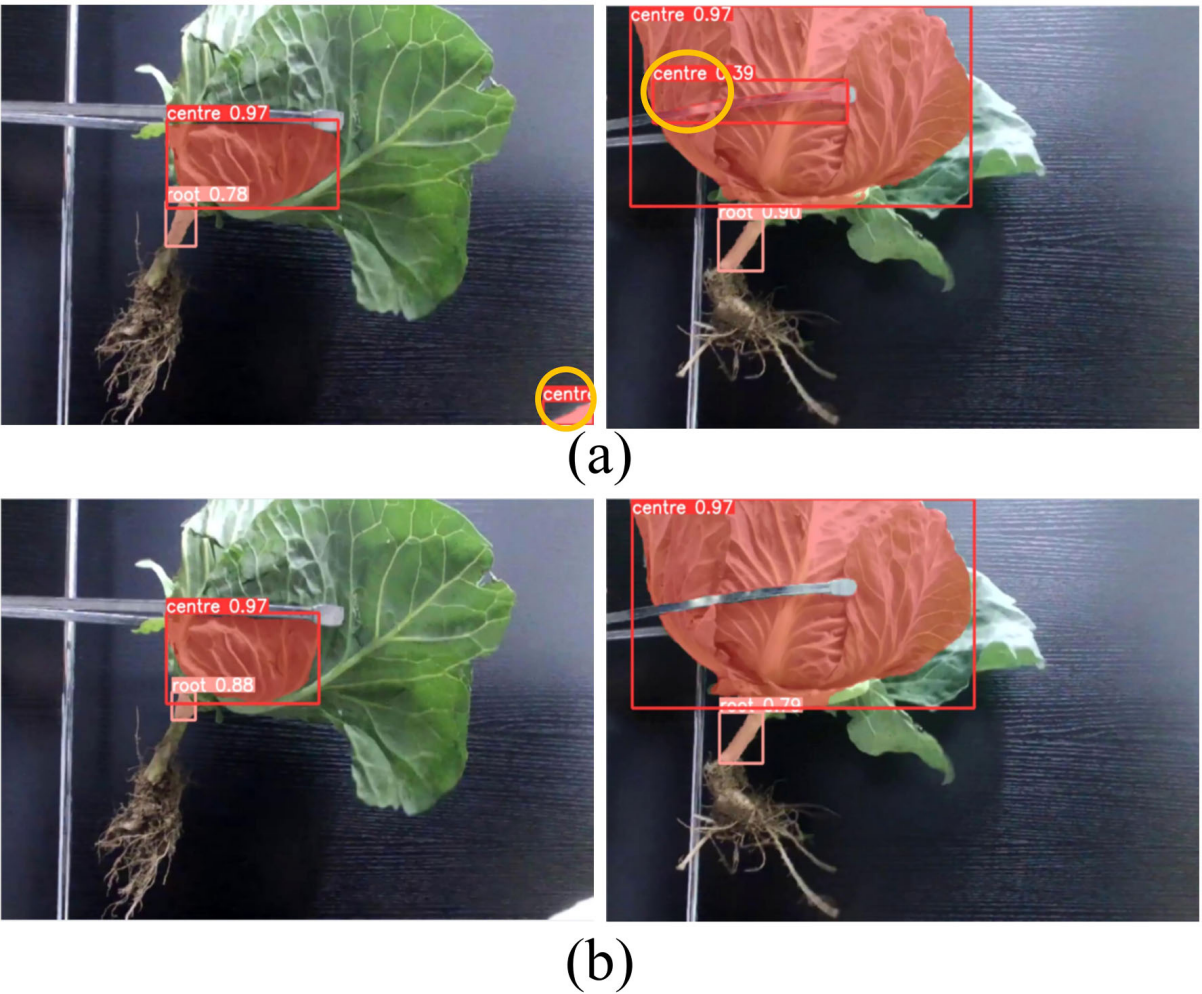
Comparison of model detection results before and after algorithm improvement. **(A)** YOLOv5s detection result, **(B)** YOLOv5-POS detection result.

To further evaluate the performance of the model, this study assessed the trained model on a test set. **Figure 14** illustrates the comparison of algorithm performance before and after enhancement. **Figure 14A** shows the detection results of the baseline YOLOv5s model, where certain areas exhibit significant false detections. In contrast, **Figure 14B** displays the detection results of the YOLOv5s-POS model, where the same areas show a marked reduction in false detections. The results indicate the YOLOv5s-POS model achieves higher detection accuracy and significantly reduces false detections.

### 3.3 Comparison of different detection algorithms

To further validate the effectiveness of the proposed model, several mainstream multi-task network and instance segmentation models were trained and evaluated using consistent training and validation datasets. The models considered for comparison encompass multi-task networks such as YOLOv5s, Mask-RCNN, Cascade-Mask-RCNN, and HTC, alongside instance segmentation models like SOLOv2 and YOLOv5-POS. The experimental results are presented in **Table 3**.

**Table 3:** Comparison of detection models.

Model	Box		Mask		Size (MB)	FPS
	mAP@0.5	mAP@0.5:0.95	mAP@0.5	mAP@0.5:0.95		
YOLOv5s	93.0%	81.1%	92.7%	79.9%	15.1	286
Mask-Rcnn	89.6%	77.2%	87.9%	70.9%	343	17
Cascade-Mask-Rcnn	88.0%	72.6%	89.6%	73.5%	588	14
HTC	88.9%	74.3%	90.1%	75.2%	589	9
SOLOv2	–	–	89.5%	74.8%	353	16
YOLOv8n	92.2%	76.9%	92.6%	76.7%	6.8	322
YOLOv8s	93.8	84.4	93.6	82.6	23.8	258
YOLOv5-POS	93.5%	81.4%	93.3%	80.1%	19.4	263

**Table 3** presents a comparative analysis of various models based on multiple metrics, including model size and frames per second (FPS). The YOLOv5s and YOLOv8n models stand out for their compact size and high processing speed, though their accuracy falls short compared to the YOLOv5-POS model. In contrast, models like Mask-RCNN, Cascade-Mask-RCNN, HTC, and SOLOv2 exhibit inferior performance in terms of model size, processing speed, and detection rates when compared to YOLOv5-POS. Although YOLOv8s achieves a marginally higher accuracy (0.3%) than YOLOv5-POS, it comes with a 20% increase in model weight. In the Box (mAP@50) and Box (mAP@50:95) metrics, YOLOv5-POS attained impressive scores of 93.5% and 81.4%, respectively, and excelled in the Mask (mAP@50) and Mask (mAP@50:95) metrics with scores of 93.3% and 80.1%, respectively. Consequently, the YOLOv5-POS model demonstrates superior performance in practical deployment scenarios.

The experimental findings underscore the substantial enhancement in accuracy achieved by the YOLOv5-POS model for object detection and instance segmentation tasks. Specifically tailored for detecting cabbage heads and roots, the YOLOv5-POS model effectively rectifies misidentifications that were evident in the original YOLOv5s model. This improvement is pivotal for accurate cabbage pose recognition predictions. Despite an increase in the number of model parameters, this study demonstrates that the proposed model not only enhances accuracy but also optimizes computational efficiency by reducing GFLOPs during the calculation process. Consequently, the model achieves efficient computations, crucial for real-time applications. Moreover, the YOLOv5-POS model boasts a compact size of 19.4 MB and a high processing speed of 263 FPS, striking an optimal balance between model size and speed. These attributes collectively contribute to delivering precise and reliable detection results.

### 3.4 Comparison of different measurement methods

To verify the accuracy and reliability of the proposed method in this study, a comparative experiment was conducted with other traditional image processing methods. The experiment utilized 138 images of three different cabbage varieties. The dataset included challenging samples with occluded, severely tilted, short, distorted, and damaged cabbage roots.

**Figure 15:**
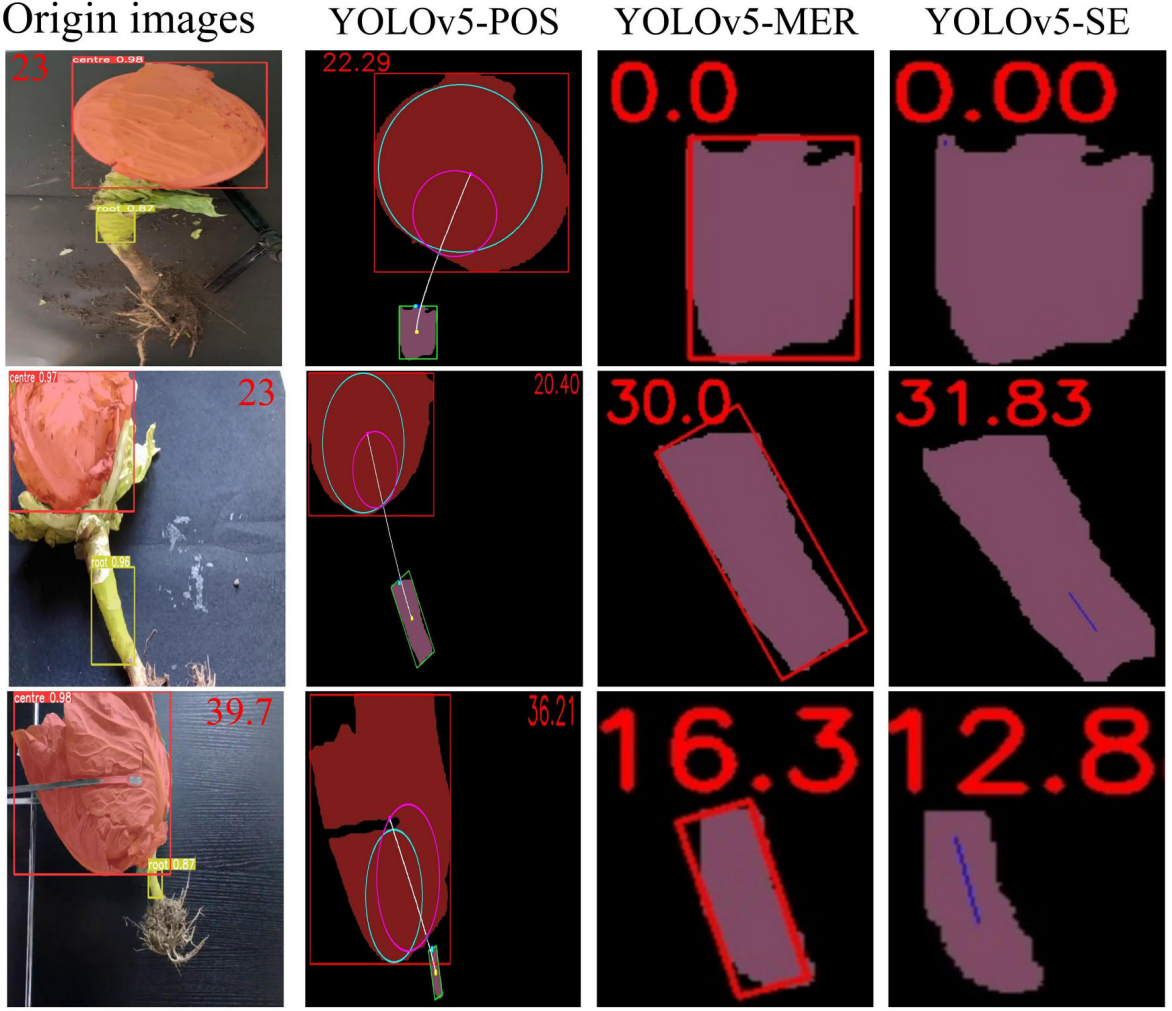
Comparison of pose angle predictions under different methods for various varieties and conditions. YOLOv5-POS is the method proposed in this study (The blue circle represents the maximum contour, and the purple circle represents the minimum contour). YOLOv5-MER combined with minimum external rectangle and YOLOv5-SE combined with skeleton extraction.

Prior to computer-based image measurements, actual angles were measured using an electronic angle measuring instrument for testing and comparison purposes. The results are presented in **Table 4** and **Figure 15**. The test accuracy of the proposed YOLOv5-POS method surpasses that of the YOLOv5-MER and YOLOv5-SE methods across the Kunming-cabbage, Harbin-cabbage, and Huzhou-cabbage datasets. Specifically, on the Harbin-cabbage dataset, the YOLOv5-POS method achieves an RMAE (Root Mean Absolute Error) of only 0.63°, indicating the highest test accuracy among the methods evaluated. On the Kunming-Cabbage dataset, although the RMAE is 2.65°, it significantly outperforms the 8.09° of the YOLOv5-MER method and the 17.50° of the YOLOv5-SE method. When considering the aggregate results across all datasets, the YOLOv5-POS method achieves a total RMAE of 1.38° and a total RMSE (Root Mean Squared Error) of 2.32, both metrics significantly better than those of the other two methods. Specifically, YOLOv5-MER records a total RMAE of 9.95° and a total RMSE of 13.32, while YOLOv5-SE records a total RMAE of 14.4° and a total RMSE of 15.2. These findings underscore the superior test accuracy of the YOLOv5-POS method when evaluated across all test datasets.

**Table 4:** Comparison table of different measurement methods.

Species Methods		Kunming-cabbage		Harbin-cabbage		Huzhou-cabbage		Sum	
		RMAE (°)	RMSE (°)	RMAE (°)	RMSE (°)	RMAE (°)	RMSE (°)	RMAE (°)	RMSE (°)
YOLOv5-POS	2.65	4.75	0.63	1.04	0.85	1.16	1.38	2.32	YOLOv5-POS
YOLOv5-ME	8.09	9.95	8.84	13.43	12.94	16.59	9.95	13.32	YOLOv5-ME
YOLOv5-SE	17.50	10.57	10.95	15.92	14.77	19.12	14.4	15.2	YOLOv5-SE

YOLOv5-POS is the method proposed in this study. YOLOv5-MER combined with minimum external rectangle and YOLOv5-SE combined with skeleton extraction.

## 4 Discussion

In addition to the proposed method, two techniques from other fields were selected for comparison based on their similarity to the technical processing object and the morphological structure of cabbage roots. These techniques include the minimum bounding rectangle method used by Guo et al. (2020) for cluster pepper identification and the skeleton extraction method used by Qi et al. (2022) for litchi trunk identification.

**Figure 16:**
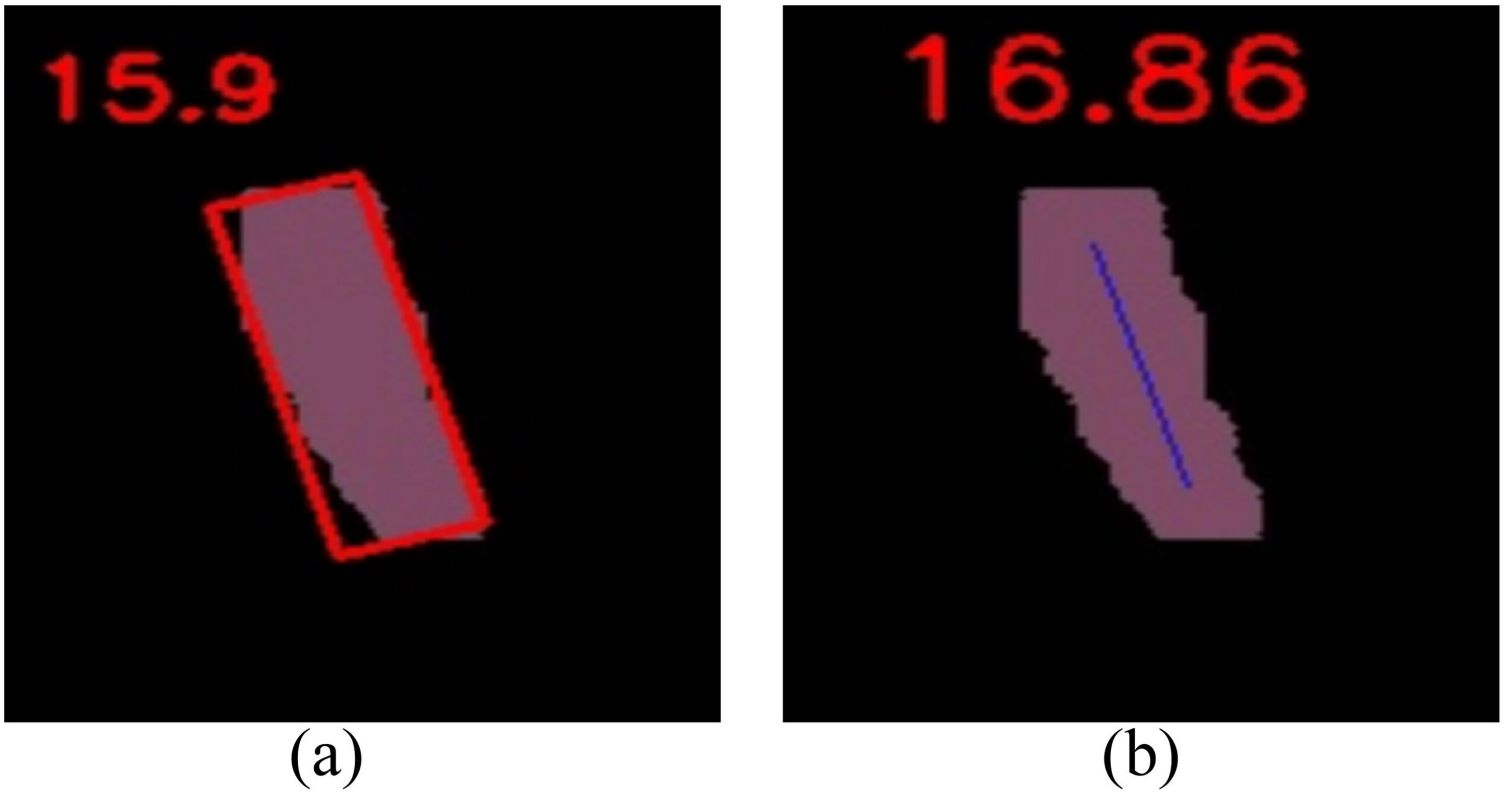
Calculation of tilt values of cabbage by different image processing methods. **(A)** Minimum enclosing rectangle, **(B)** Skeleton extraction.

**Figure 17:**
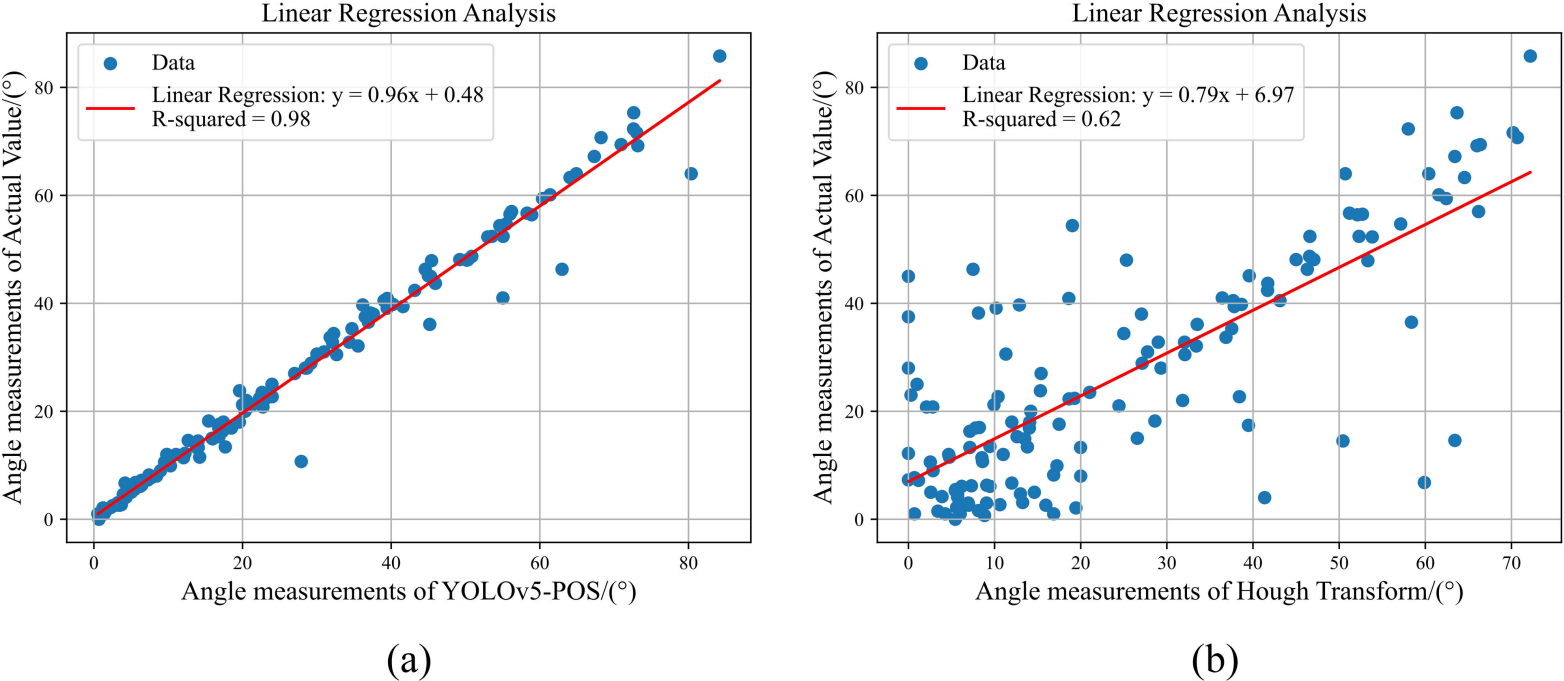
The linear regression analysis results of the different methods. **(A)** YOLOv5-POS, **(B)** Hough Transform.

As shown in **Figure 16**, when external leaf occlusion is absent, the traditional skeleton extraction and bounding box approaches exhibit root tilt prediction performance comparable to the method proposed in this study. However, the limitations of these conventional methods become evident under conditions of external leaf occlusion. The skeleton extraction method is particularly vulnerable to noise interference and has high computational complexity, hindering its ability to accurately capture the true root structure. Similarly, the bounding box method struggles with the complex shapes and varying postures of cabbage roots, failing to fully represent the internal structure, which results in a significant decline in detection accuracy. In contrast, the method proposed in this study demonstrates enhanced robustness and accuracy in the presence of external leaf occlusion. By integrating key point localization with Bezier curve modeling, it effectively addresses the challenges posed by partially occluded roots, achieving more precise measurement of root tilt angles. As shown in **Figure 17**, experimental results indicate that YOLOv5-POS achieves an absolute error of approximately 1° in root angle prediction, with an accuracy rate of 98%. Compared to other methods, the approach proposed in this study exhibits superior performance in prediction root posture.

Moreover, the prediction model integrates the KD-Tree algorithm to enhance its prediction speed. To comprehensively assess the efficacy of the KD-Tree algorithm, this study conducts comparative experiments. The KD-Tree algorithm is employed during the search for nearest neighbor points to optimize search efficiency. The comparison results reveal a reduction in test time from 111 milliseconds to 28 milliseconds following the integration of the KD-Tree algorithm, resulting in a nearly fourfold increase in speed.

While the prediction method proposed in this study has achieved notable accuracy, it still has inherent limitations. The current dataset, though encompassing images with varying illumination levels, lacks samples under high lighting conditions. This limitation reduces the adaptability of the model to different cabbage varieties and real-world scenarios. In high-light environments, overexposure can lead to detail loss, impairing root recognition and angle prediction. Additionally, the increase in parameters and weights required for enhanced model accuracy complicates its deployment and application. Future research should focus on acquiring image data of Chinese cabbage at different growth stages, captured from multiple angles and under diverse lighting conditions. An expanded dataset will improve the robustness of the model against environmental interference, enhance its generalization ability, and support the design of an effective light-shielding structure to mitigate the effects of exposure during imaging. Furthermore, the application of lightweight techniques such as pruning and distillation will aid in optimizing the model to better meet the demands of complex and sophisticated applications.

## 5 Conclusion

To effectively address the challenges of cabbage pose recognition in complex environments, this study introduces an innovative YOLOv5-POS model. Based on YOLOv5s, the model focuses on the precision detection and segmentation of cabbage heads and roots. By optimizing the segmentation head module and incorporating an improved C-RepGFPN model to replace the traditional Neck layer, the study significantly enhances the detection accuracy and efficiency of the model. Additionally, the study creatively applies the Bezier curve construction method for predicting the posture of cabbage root, achieving precision predictions even under completely leaf occlusions. The experimental results validate the good performance of the YOLOv5-POS model. The model achieves a detection accuracy of 93.3% and an F1 score of 98.8% across various cabbage varieties. In segmentation tasks, it reaches scores of 93.5% and 81.4% for mAP@0.5 and mAP@0.5:0.95, respectively. These results demonstrate the advantages of theYOLOv5-POS model in accuracy and reliability. By accurately locating key points and applying Bezier curves, the model can precisely predict the growth path of cabbage root systems, with a root angle testing accuracy of 98% and a feature extraction to decision processing time of just 28 milliseconds, fully meeting real-time detection requirements. Furthermore, the YOLOv5-POS model proposed in this study significantly outperforms existing mainstream segmentation detection models and measurement methods in balancing accuracy and deployment efficiency. It demonstrates exceptional real-time detection and deployment capabilities on mobile devices, providing robust technical support for cabbage harvest decisions.

## Data Availability

The raw data supporting the conclusions of this article will be made available by the authors, without undue reservation.

## References

[B1] Dai J. Li Y. He K. Sun J. (2016). R-fcn: object detection via region-based fully convolutional networks. Adv. Neural Inf. Process. systems. 29. doi: 10.48550/arXiv.1605.06409

[B2] Dongdong D. Jun W. Shanshan Q. (2015). Analysis and test of splitting failure in the cutting process of cabbage root. Int. J. Agric. Biol. Engineering. 8, 27–34. doi: 10.3965/j.ijabe.20150804.1723

[B3] Guo Y. Zhang Y. Chen Z. Li F. (2020). Cluster prickly ash image recognition and picking point location based on multistage image transformation and growth characteristics. Int. J. Res. Agric. Forestry. 7, 28–34.

[B4] Hua X. Li H. Zeng J. Han C. Chen T. Tang L.. (2023). A review of target recognition technology for fruit picking robots: from digital image processing to deep learning. Appl. Sci. 13, 4160. doi: 10.3390/app13074160

[B5] Hussain M. He L. Schupp J. Lyons D. Heinemann P. (2023). Green fruit segmentation and orientation estimation for robotic green fruit thinning of apples. Comput. Electron. Agriculture. 207, 107734. doi: 10.1016/j.compag.2023.107734

[B6] Jin X. Yang H. He X. Liu G. Yan Z. Wang Q. (2023). Robust lidar-based vehicle detection for on-road autonomous driving, Vol. 15. *Remote Sensing* (Basel, Switzerland: MDPI).

[B7] Lee T. Seok J. (2023). “Multi task learning: A survey and future directions,” in 2023 International Conference on Artificial Intelligence in Information and Communication (ICAIIC). (Bali, Indonesia: IEEE), 232–235. doi: 10.1109/ICAIIC57133.2023.10067098

[B8] Liu Q. Gong X. Li J. Wang H. Liu R. Liu D.. (2023). A multitask model for realtime fish detection and segmentation based on yolov5. PeerJ Comput. Sci. 9, e1262. doi: 10.7717/peerj-cs.1262 PMC10280594 37346717

[B9] Luo L. Tang Y. Lu Q. Chen X. Zhang P. Zou X. (2018). A vision methodology for harvesting robot to detect cutting points on peduncles of double overlapping grape clusters in a vineyard. Comput. industry. 99, 130–139. doi: 10.1016/j.compind.2018.03.017

[B10] Mingxuan Z. Wei L. Hui L. Ruinan Z. Yiming D. (2022). Anti-gravity stem-seeking restoration algorithm for maize seed root image phenotype detection. Comput. Electron. Agriculture. 202, 107337. doi: 10.1016/j.compag.2022.107337

[B11] Ogedengbe T. C. Malomo O. J. Akanji N. E. (2022). Post-harvest losses and reduction techniques in crop production: A review. Int. J. Of Agric. Science Res. And Technol. In Extension And Educ. Systems. 12, 225–233. doi: 20.1001.1.22517588.2022.12.4.5.0

[B12] Qi X. Dong J. Lan Y. Zhu H. (2022). Method for identifying litchi picking position based on yolov5 and pspnet. Remote Sensing. 14, 2004. doi: 10.3390/rs14092004

[B13] Ronneberger O. Fischer P. Brox T. (2015). “U-net: convolutional networks for biomedical image segmentation,” in Medical image computing and computer-assisted intervention–MICCAI 2015: 18th international conference, Munich, Germany, October 5-9, 2015, proceedings, part III 18. (Cham: Springer), 234–241.

[B14] Roy A. M. Bhaduri J. (2022). Real-time growth stage detection model for high degree of occultation using densenet-fused yolov4. Comput. Electron. Agriculture. 193, 106694. doi: 10.1016/j.compag.2022.106694

[B15] Sa I. Ge Z. Dayoub F. Upcroft B. Perez T. Mccool C. (2016). Deepfruits: A fruit detection system using deep neural networks. Sensors. 16 (8), 1222. doi: 10.3390/s16081222 27527168 PMC5017387

[B16] Sanchez-Reyes J. (2020). Comment on “Defining a curve as a bezier curve. J. Taibah Univ. Science. 14, 849–850. doi: 10.1080/16583655.2020.1780057

[B17] Sapkota R. Ahmed D. Karkee M. (2024). Comparing yolov8 and mask R-cnn for instance segmentation in complex orchard environments. Artif. Intell. Agriculture. 13, 84–99. doi: 10.1016/j.aiia.2024.07.001

[B18] Sun T. Zhang W. Miao Z. Zhang Z. Li N. (2023). Object localization methodology in occluded agricultural environments through deep learning and active sensing. Comput. Electron. Agriculture. 212, 108141. doi: 10.1016/j.compag.2023.108141

[B19] Tian F. Hu G. Yu S. Wang R. Song Z. Yan Y.. (2023). An efficient multi-task convolutional neural network for dairy farm object detection and segmentation. Comput. Electron. Agriculture. 211, 108000. doi: 10.1016/j.compag.2023.108000

[B20] Tong W. Zhang J. Cao G. Song Z. Ning X. (2023). Design and experiment of a low-loss harvesting test platform for cabbage. Agriculture. 13, 1204. doi: 10.3390/agriculture13061204

[B21] Wang C.-Y. Bochkovskiy A. Liao H.-Y. M. (2023). “Yolov7: trainable bag-of-freebies sets new state-of-the-art for real-time object detectors,” in Proceedings of the IEEE/CVF Conference on Computer Vision and Pattern Recognition. (Vancouver, BC, Canada: IEEE), 7464–7475.

[B22] Wu F. Duan J. Chen S. Ye Y. Ai P. Yang Z. (2021). Multi-target recognition of bananas and automatic positioning for the inflorescence axis cutting point. Front. Plant Sci. 12. doi: 10.3389/fpls.2021.705021 PMC8592935 34795680

[B23] Xiong J. Lin R. Liu Z. He Z. Tang L. Yang Z.. (2018). The recognition of litchi clusters and the calculation of picking point in a nocturnal natural environment. Biosyst. Engineering. 166, 44–57. doi: 10.1016/j.biosystemseng.2017.11.005

[B24] Yamamoto K. Guo W. Yoshioka Y. Ninomiya S. (2014). On plant detection of intact tomato fruits using image analysis and machine learning methods. Sensors. 14, 12191–12206. doi: 10.3390/s140712191 25010694 PMC4168514

[B25] Ying Y. Jing H. Tao Y. Jin J. Ibarra J. G. Chen Z. (2000). Application of machine vision in inspecting stem and shape of fruits. Biol. Qual. Precis. Agric. II. 4203, 122–130. doi: 10.1117/12.411746

[B26] Yu Y. Zhang K. Yang L. Zhang D. (2019). Fruit detection for strawberry harvesting robot in non-structural environment based on mask-rcnn. Comput. Electron. Agriculture. 163, 104846. doi: 10.1016/j.compag.2019.06.001

[B27] Zhao Z. Hicks Y. Sun X. Luo C. (2023). Peach ripeness classification based on a new one-stage instance segmentation model. Comput. Electron. Agriculture. 214, 108369. doi: 10.1016/j.compag.2023.108369

[B28] Zhaoxin G. Han L. Zhijiang Z. Libo P. (2022). Design a robot system for tomato picking based on yolo V5. IFAC-PapersOnLine. 55, 166–171. doi: 10.1016/j.ifacol.2022.05.029

[B29] Zhu L. Zhang Z. Lin G. Chen P. Li X. Zhang S. (2023). Detection and localization of tea bud based on improved yolov5s and 3d point cloud processing. Agronomy. 13, 2412. doi: 10.3390/agronomy13092412

